# Glioma cell VEGFR-2 confers resistance to chemotherapeutic and antiangiogenic treatments in PTEN-deficient glioblastoma

**DOI:** 10.18632/oncotarget.2910

**Published:** 2015-02-19

**Authors:** Tobias Kessler, Felix Sahm, Jonas Blaes, Matthias Osswald, Petra Rübmann, David Milford, Severino Urban, Leonie Jestaedt, Sabine Heiland, Martin Bendszus, Anne Hertenstein, Philipp-Niclas Pfenning, Carmen Ruiz de Almodóvar, Antje Wick, Frank Winkler, Andreas von Deimling, Michael Platten, Wolfgang Wick, Markus Weiler

**Affiliations:** ^1^ Clinical Cooperation Unit Neurooncology, German Cancer Consortium (DKTK), German Cancer Research Center (DKFZ), Heidelberg, Germany; ^2^ Clinical Cooperation Unit Neuroimmunology and Brain Tumor Immunology and German Cancer Consortium (DKTK), German Cancer Research Center (DKFZ), Heidelberg, Germany; ^3^ Clinical Cooperation Unit Neuropathology, German Cancer Consortium (DKTK), German Cancer Research Center (DKFZ), Heidelberg, Germany; ^4^ Department of Neurooncology at the National Center for Tumor Diseases, Heidelberg University Hospital, Heidelberg, Germany; ^5^ Department of General Neurology, Heidelberg University Hospital, Heidelberg, Germany; ^6^ Department of Neuropathology, Heidelberg University Hospital, Heidelberg, Germany; ^7^ Department of Neuroradiology, Heidelberg University Hospital, Heidelberg, Germany; ^8^ Biochemistry Center Heidelberg University, Heidelberg, Germany

**Keywords:** angiogenesis, glioblastoma, invasion, *phosphatase and tensin homolog deleted on chromosome 10 (PTEN)*, vascular endothelial growth factor receptor (VEGFR)-2

## Abstract

Loss of the tumor suppressor phosphatase and tensin homolog deleted on chromosome 10 (PTEN) is a prerequisite for tumor cell-specific expression of vascular endothelial growth factor receptor (VEGFR)-2 in glioblastoma defining a subgroup prone to develop evasive resistance towards antiangiogenic treatments. Immunohistochemical analysis of human tumor tissues showed VEGFR-2 expression in glioma cells in 19% of specimens examined, mainly in the infiltration zone. Glioma cell VEGFR-2 positivity was restricted to PTEN-deficient tumor specimens. PTEN overexpression reduced VEGFR-2 expression *in vitro*, as well as knock-down of *raptor* or *rictor*. Genetic interference with VEGFR-2 revealed proproliferative, antiinvasive and chemoprotective functions for VEGFR-2 in glioma cells. VEGFR-2-dependent cellular effects were concomitant with activation of 'kappa-light-chain-enhancer’ of activated B-cells, protein kinase B, and N-myc downstream regulated gene 1. Two-photon *in vivo* microscopy revealed that expression of VEGFR-2 in glioma cells hampers antiangiogenesis. Bevacizumab induces a proinvasive response in VEGFR-2-positive glioma cells. Patients with PTEN-negative glioblastomas had a shorter survival after initiation of bevacizumab therapy compared with PTEN-positive glioblastomas. Conclusively, expression of VEGFR-2 in glioma cells indicates an aggressive glioblastoma subgroup developing early resistance to temozolomide or bevacizumab. Loss of PTEN may serve as a biomarker identifying those tumors upfront by routine neuropathological methods.

## INTRODUCTION

Agents targeting the vascular endothelial growth factor (VEGF)/VEGF receptor (VEGFR)-2 axis in glioblastoma have widely been tested [1]. However, recent phase III trials in newly diagnosed glioblastoma demonstrated a failure of the monoclonal anti-VEGF-directed antibody bevacizumab (BEV) to extend overall survival (OS) when combined with chemoradiation, despite benefits in progression-free survival (PFS) and quality of life [2, 3]. One potential reason for the lack of an overall survival benefit is that angiogenesis inhibitors, by impairment of tumor angiogenesis, may eventually have detrimental effects, including induction of enhanced tumor cell invasion into surrounding tissue in yet to be defined subgroups [4–6]. In clinical series, upon progression on BEV, glioblastoma can display a more infiltrative pattern of recurrence [7]. Yet, a recent study from our own group argued that the promotion of distant tumor growth or a gliomatosis-like growth phenotype at recurrence is relevant at best in a minority of patients [8]. An analysis from the AVAglio trial also argues against a specific propensity of BEV to induce diffuse or infiltrative growth, but identified a subgroup of patients showing this tumor growth behavior [9]. Hence, molecular profiling allowing an identification of subgroups of tumors that are at increased risk to develop early resistance towards antiangiogenic treatments remains an unmet need.

According to the molecular subtypes of Phillips *et al*. loss of the phosphatase and tensin homolog deleted on chromosome 10 (PTEN) and expression of VEGFR-2 are features of the mesenchymal glioblastoma phenotype [10]. Mutations or deletions in the *PTEN* gene commonly lead to activation of the phosphoinositide 3-kinase (PI3K)/protein kinase B (AKT/PKB)/mammalian target of rapamycin (mTOR) signaling network and have previously been reported to be associated with reduced survival of glioma patients [11]. Recently, a molecular mechanism was proposed by which ablation of the VEGF/VEGFR-2 signaling cascade increases activity of the hepatocyte growth factor (HGF) receptor MET through a MET/VEGFR-2 heterocomplex and thus promotes tumor cell invasion in glioblastoma [6], although clinical evidence for an effect of MET inhibition in patients with glioblastoma is lacking. Furthermore, expression of VEGFR-2 by tumor cells in addition to its constitutive presence on endothelial cells in glioblastoma has been controversial, though there has been increasing evidence for a restricted expression of VEGFR-2 in a subset of tumor cells [12–16].

The goal of the present work was to validate the expression of VEGFR-2 in glioblastoma cells and tissues with respect to the PTEN status and to characterize VEGFR-2-specific functions in glioma cells focusing on clinically relevant therapeutic modalities.

## RESULTS

### A subgroup of glioblastoma exhibits tumor cell expression of VEGFR-2, predominantly in the infiltration zone

**Figure 1:**
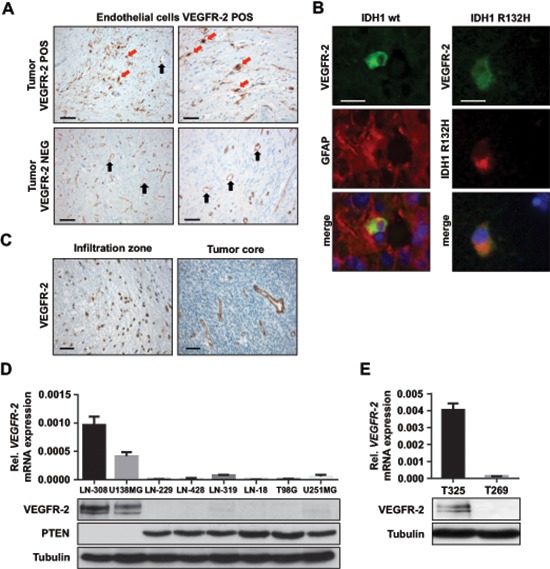
VEGFR-2 is expressed by tumor cells in a subset of glioblastoma **(A)** Immunohistochemical analysis of VEGFR-2 expression in primary, i.e., IDH1 wild-type glioblastoma. The upper two images show tumor tissue with VEGFR-2 expression in both endothelial and tumor cells. The lower two images depict a tumor with VEGFR-2 expression confined only to endothelial cells. VEGFR-2-positive tumor cells are indicated by red arrows, endothelial cells are marked by black arrows. Scale bars on left images, 100 μm; scale bars on right images, 50 μm. **(B)** Immunoflourescence of patient-derived glioblastoma cryosections. Left column: Primary, i.e., IDH1 wild-type glioblastoma that shows positive co-immunostaining for VEGFR-2 and GFAP within the same cell proving tumor cell-specific expression of VEGFR-2. Right column: Secondary glioblastoma harboring the IDH1R132H mutation that displays positive co-immunostaining for VEGFR-2 and the mutated IDH1 protein indicating expression of VEGFR-2 in tumor cells. Scale bars, 20 μm. **(C)** VEGFR-2-specific IHC of a glioblastoma showing the infiltration zone with VEGFR-2-positive tumor cells (left) and a tumor core with VEGFR-2 expression confined to the vasculature (right). Scale bars, 20 μm. **(D)** VEGFR-2 and PTEN expression in eight human glioma cell lines. Upper panel: qRT-PCR analysis, *VEGFR-2* mRNA is displayed relative to *actin* expression. Lower panel: Immunoblot analysis for VEGFR-2 and PTEN, tubulin served as a loading control. **(E)** VEGFR-2 expression in two GICs. Upper panel: qRT-PCR analysis, *VEGFR-2* mRNA is displayed relative to *actin* expression. Lower panel: immunoblot analysis, tubulin served as a loading control.

Aiming to assess the incidence of tumoral VEGFR-2 expression, we evaluated the expression pattern of VEGFR-2 in a total of 106 patient-derived glioblastoma specimens. As expected, endothelial cells in all of these tumor tissues exhibited strong immunoreactivity for VEGFR-2. Yet, in 20 of the 106 specimens (19%), VEGFR-2 expression was additionally found to be confined to tumor cells (Figure 1A; Figure S1A, S1B). To verify expression of VEGFR-2 specifically on glioma cells, we used a co-staining with the tumor cell-specific IDH1^R132H^ antibody (Figure 1B). Moreover, subgroup analysis of 40 specimens allowing a distinct differentiation between tumor core (*n* = 34) and infiltration zone (*n* = 6) disclosed that VEGFR-2-positive glioblastoma cells were more frequently found in the infiltration zone. Three of the six glioblastoma specimens (50%) of which the infiltration zones were assessable showed VEGFR-2 expression only there, whereas from the other 34 tumors only two demonstrated VEGFR-2 expression in the tumor core (5.9%, *p* = 0.018, exact Fisher test; Figure 1C). Taken together, next to its known vessel-bound expression, VEGFR-2 is additionally expressed by glioblastoma cells, preferentially in the tumor infiltration zone.

### Loss of PTEN and activated PI3K/AKT/mTOR signaling are required for expression of VEGFR-2 in glioblastoma cells

Two of eight glioma cell lines and one of two GIC with known PTEN status expressed VEGFR-2 mRNA and protein (Figure 1D, 1E). Expression data were confirmed by immunofluorescence and flow cytometry (Figure S2A, S2B). A comparison between VEGFR-2 expression and PTEN status showed expression for VEGFR-2 only in cells with deficiency of PTEN, indicating a mutually exclusive expression pattern for VEGFR-2 and PTEN in glioma cell lines and GIC cultures (Table S1). Treatment of VEGFR-2-positive LN-308 glioma cells with exogenous VEGF (50 ng/ml) led to increased phosphorylation of VEGFR-2, p90RSK and AKT, confirming active VEGFR-2 signaling in these cells *in vitro* (Figure S2C).

**Figure 2:**
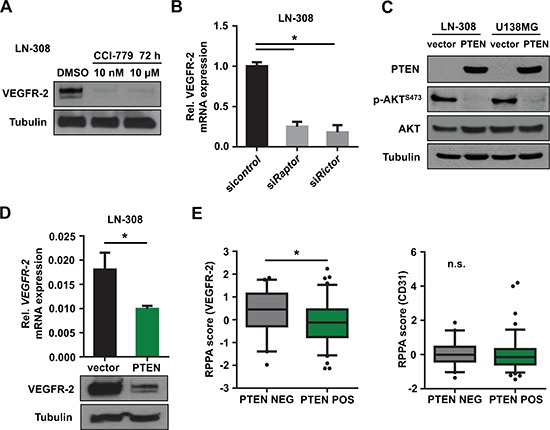
Inhibition of the PI3K/AKT/mTOR pathway reduces tumoral VEGFR-2 expression **(A)** Immunoblot analysis of lysates derived from glioma cell lines following treatment with CCI-779 at indicated concentrations. Tubulin served as a loading control. **(B)** qRT-PCR analysis for *VEGFR-2* mRNA expression relative to *actin* after siRNA-mediated knock-down of *raptor* (constituent of mTORC1) and *rictor* (constituent of mTORC2), respectively. **(C)** Immunoblot analysis of LN-308 glioma cells with stable transfection of PTEN or empty vector control. Phospho-AKT^S473^ was used to monitor PTEN activity. Tubulin served as a loading control. **(D)** qRT-PCR and immunoblot analysis for VEGFR-2 expression in PTEN-overexpressing LN-308 cells compared with controls. Tubulin served as a loading control. **(E)** TCGA database analysis of glioblastoma specimens. Specimens with mutation and/or homozygous deletion of *PTEN* were denoted as ‘PTEN NEG’, specimens without *PTEN* mutation or homozygous deletion as ‘PTEN POS’. VEGFR-2 (left) and CD31 (right) reverse phase protein array (RPPA) score is shown depending on the PTEN status.

Due to its activation upon loss of PTEN, mTOR might govern the expression of VEGFR-2. Pharmacologic inhibition of mTOR using CCI-779 (temsirolimus) depleted VEGFR-2 protein levels in PTEN-deficient LN-308 glioma cells (Figure 2A). Moreover, selective inactivation of either mTORC1 or mTORC2 by RNAi-mediated knock-down of either *raptor* or *rictor* caused a robust decrease in *VEGFR-2* mRNA expression suggesting that both mTOR complexes are required for VEGFR-2 expression (Figure 2B). Exogenous expression of PTEN in PTEN-deficient and VEGFR-2-positive LN-308 and U138MG glioma cells (Figure 2C) led to reduced VEGFR-2 mRNA and protein levels (Figure 2D), confirming a VEGFR-2-suppressive effect for PTEN. In line with previous reports, modulation of PTEN expression confirmed robust antiinvasive and anticlonogenic properties for PTEN in several glioma cell lines (Figure S3A–S3D).

Next, we wanted to know whether the interdependence between PTEN loss and VEGFR-2 expression in glioma cells can be confirmed in patient-derived tumor tissues. Immunohistochemistry (IHC) for both proteins in a series of 79 glioblastoma tissues demonstrated that of these, 54 tumors were either positive for PTEN or tumoral VEGFR-2 expression, and 25 tumors were negative for both markers (Table 1). Importantly, we did not find a single PTEN-positive glioblastoma containing VEGFR-2-positive tumor cells (*p* < 0.001, exact Fisher test; Table 1), further supporting our hypothesis that tumoral VEGFR-2 expression requires loss of PTEN.

**Table 1:** Correlation between PTEN status and VEGFR-2 expression in a series of 79 human glioblastoma specimens Protein expression of PTEN and VEGFR-2 was determined by IHC. Only glioblastomas clearly showing VEGFR-2 expression in tumor cells (in addition to its expression on endothelial cells) were evaluated as VEGFR-2-positive.

	PTEN POS	PTEN NEG	*p*
Total [*n]*	37	42	
VEGFR-2 POS [*n*], (%)	0 (0)	17 (40)	**< 0.001**
VEGFR-2 NEG [*n*], (%)	37 (100)	25 (60)	

**Abbreviations:** POS, positive; NEG, negative.

PTEN IHC does not necessarily reflect genetic alterations in the *PTEN* gene possibly leading to translation of the full-length but yet dysfunctional PTEN protein. Hence, we completed our study by sequencing and multiplex ligation-dependent probe amplification (MLPA) of the *PTEN* gene in 28 of the 79 glioblastoma specimens (35%). This demonstrated *PTEN* mutations in four (by sequencing) and deletions in all 12 tumors (by MLPA) that were tested PTEN-negative on IHC. Conversely, 16 tumors assessed as PTEN-positive on IHC revealed mutations in only two cases and deletions in 12 additional cases (the latter assumingly being functional heterodeletions).

Analysis of glioblastoma tissues from The Cancer Genome Atlas (TCGA) database [17] demonstrated increased VEGFR-2 protein expression determined with reverse phase protein array in tumors with either copy number variations (CNV) or mutations in the *PTEN* gene compared with tumors without genetic alterations. Of note, CD31 expression as a marker for endothelial cells was not affected by PTEN alterations (Figure 2E).

### Tumor cell expression of VEGFR-2 drives glioma cell proliferation and clonogenicity

To learn more about the intrinsic functions of VEGFR-2 expression in tumor cells, we generated specific sh*VEGFR-2* transfectants of LN-308 and U138MG glioma cells as well as stably VEGFR-2-overexpressing LN-229 glioma cells (Figure S4). Available data on VEGFR-2 and its effects on glioma cell proliferation are controversial [15, 16]. In our hands, *VEGFR-2* knock-down in LN-308 and U138MG glioma cells caused significantly decreased proliferation compared with control cells (Figure S5A). Pharmacological ablation of VEGFR-2 activity through treatment with AZD2171 (Cediranib) demonstrated a similar effect (Figure S5B). *Vice versa*, overexpression of VEGFR-2 in LN-229 cells increased proliferation and made these cells susceptible to treatment with AZD2171 that decreased proliferation of these cells significantly (Figure S5C). Moreover, LN-308 and U138MG sh*VEGFR-2* cells revealed impaired clonogenic survival (Figure S5D). To further assess these VEGFR-2-dependent effects in conditions that better resemble the three-dimensional brain tissue cytoarchitecture *in vivo*, we set up an experimental model system using organotypic brain slice cultures (Figure S6A, S6B). Consistent with our observations *in vitro*, LN-308 sh*control* cells implanted in brain slices showed a higher proliferation rate than sh*VEGFR-2* cells (Figure S6C), supporting the view of a proproliferative function of VEGFR-2 in glioma cells.

### Tumor cell expression of VEGFR-2 increases resistance of glioma cells to TMZ

**Figure 3:**
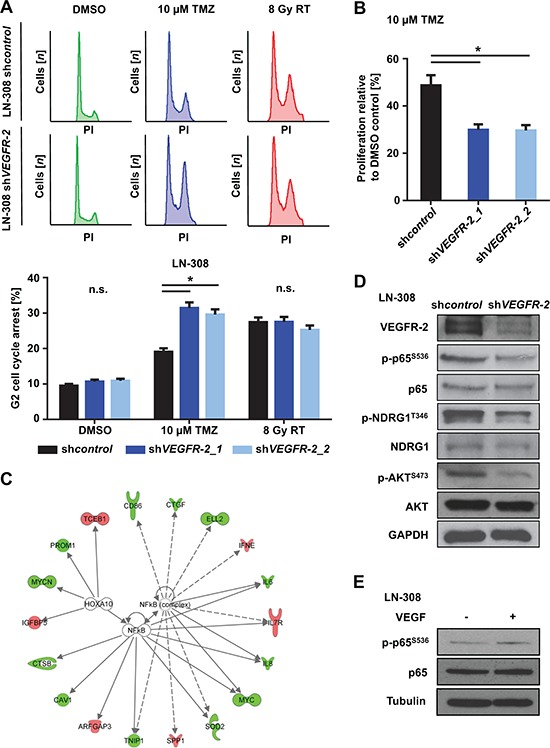
VEGFR-2 promotes resistance to alkylating chemotherapy and activates NF-κB-dependent signaling pathways **(A)** Upper panel: LN-308 sh*control* and sh*VEGFR-2* cells were treated with DMSO or TMZ (10 μM) for 72 h or radiotherapy (8 Gy) and analyzed by flow cytometry after staining with propidium iodide (PI). Lower panel: G2-arrested cells compared with all cells in each respective sample after indicated treatment and analysis by PI flow cytometry. **(B)** RTCA proliferation assay of LN-308 sh*control* and sh*VEGFR-2* cells treated with TMZ (10 μM) or equivalent DMSO for 72 h prior to the initiation of the assay. Proliferation was assessed for six days after finishing TMZ treatment. **(C)** of transcription factor analysis showing data from the top 300 deregulated genes in the whole-genome mRNA microarray. LN-308 sh*control vs*. sh*VEGFR-2* and U138MG sh*control vs*. sh*VEGFR-2* cells were analyzed. IPA connects dysregulated downstream genes of transcription factors and predicts activation or inactivation of involved transcription factors by comparison of deregulated downstream genes. Data for NF-κB complex and HOXA10 are shown. Downregulation of target genes in response to *VEGFR-2* knock-down is indicated in green, upregulation in red. GAPDH served as a loading control. **(D)** Immunoblot analysis for VEGFR-2, phospho-p65^S536^, p65, phospho-NDRG1^T346^, NDRG1, phospho-AKT^S473^ and AKT protein expression in sh*control* and sh*VEGFR-2* cells. **(E)** Immunoblot analysis of LN-308 glioma cells stimulated with VEGF (50 ng/ml) for 5 minutes. p65- and p-p65^S536^-specific bands were quantified using ImageJ. Quantification revealed an absolute increase of 29% in p-p65^S536^ expression when comparing cells stimulated with VEGF to cells not stimulated with VEGF. The relative increase after normalizing the value of the p-p65^S536^ band to each corresponding p65 value was 27%. Tubulin served as a loading control.

Next, we analyzed potential effects of tumoral VEGFR-2 expression on clinically relevant modalities of treatment in glioblastoma, i.e., alkylating chemotherapy with temozolomide (TMZ) and radiotherapy. LN-308 and U138MG sh*VEGFR-2* cells showed a higher sensitivity to TMZ-induced G2 cell cycle arrest (Figure 3A and Figure S7), whereas sensitivity towards radiotherapy was unchanged (Figure 3A). Moreover, proliferation assays performed over six days following TMZ treatment showed a significant increase in sensitivity to TMZ in sh*VEGFR-2* cells, essentially confirming the cell cycle distribution data and indicating that VEGFR-2-positive tumor cells are more resistant to alkylating chemotherapy with TMZ (Figure 3B).

### VEGFR-2 activates NF-κB-dependent transcriptional pathways and chemoresistance factors in glioma cells

Whole-genome mRNA microarray analyses of LN-308 and U138MG sh*VEGFR-2* cells with subsequent evaluation in an Ingenuity Pathway Analysis (IPA) revealed the nuclear factor ‘kappa-light-chain-enhancer’ of activated B-cells (NF-κB) complex as the only transcription factor complex whose target genes were significantly deregulated in both cell lines upon *VEGFR-2* knock-down. Altogether, 14 target genes of the NF-κB complex were found to be regulated in response to *VEGFR-2* knock-down in LN-308 cells, whereas 23 target genes were affected in U138MG cells. The most prominent NF-κB target genes were *caveolin-1* (*CAV1*), *interleukin-6* (*IL-6*), *interleukin-8* (*IL-8*), and *the v-myc myelocytomatosis viral oncogene homolog* (*c-myc*) (Figure 3C). Immunoblot analysis of p-p65 (RELA) as an activation marker of the NFκB-complex showed increased phosphorylation of p65 in VEGFR-2-positive cells confirming the influence of VEGFR-2 on the activity of the NF-κB-complex. Knock-down of *VEGFR-2* in glioma cells led to decreased phosphorylation and thus reduced activity of two central resistance factors, protein kinase B (AKT/PKB)^S473^ and N-myc downstream regulated gene (NDRG)1^T346^ [11, 18], suggesting molecular cues for the increased resistance of VEGFR-2-positive glioma cells towards alkylating chemotherapy (Figure 3D). Moreover, stimulation with VEGF (50 ng/ml) increased phosphorylation of p65 in LN-308 wild-type cells (Figure 3E).

### Ablation of VEGFR-2 signaling in glioma cells induces a proinvasive tumor cell phenotype

**Figure 4:**
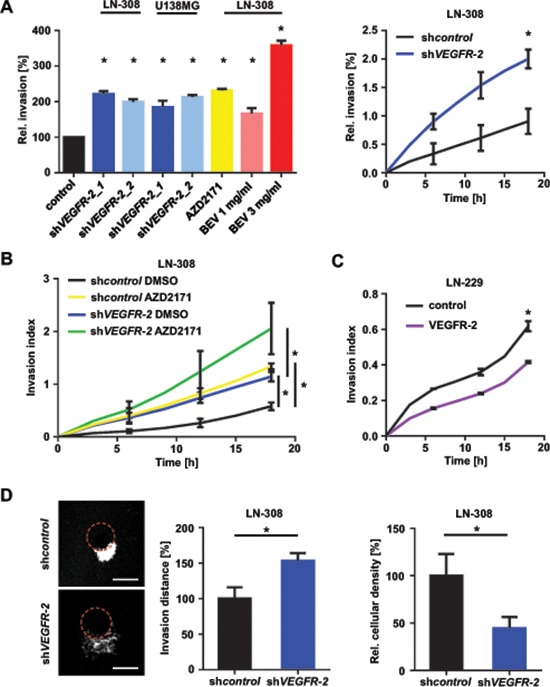
Blockade of VEGF/VEGFR-2 signaling stimulates glioma cell invasion **(A)** Overview of glioma cell invasiveness in response to different forms of inhibition and abrogation of the VEGFR-2 pathway through *VEGFR-2* knock-down or treatment with AZD2171 or BEV. Representative results of different RTCA analyses relative to appropriate controls are shown (left). Sample of the invasive kinetic of LN-308 sh*control* and sh*VEGFR-2* cells in a RTCA assay (right). **(B)** RTCA invasion assay of LN-308 sh*control* and sh*VEGFR-2* cells treated with either AZD2171 (100 nM) or equivalent DMSO. **(C)** RTCA invasion assay of LN-229 cells stably overexpressing VEGFR-2 and respective control cells transfected with the empty expression vector. **(D)** Left: Representative images of tumor invasion in the organotypic brain slice culture assay using beads coated with LN-308 sh*control* or sh*VEGFR-2* cells. Equal GFP intensity of LN-308 sh*control* and sh*VEGFR-2* cells was verified by flow cytometry before assay was started. The edge of each bead is marked by a dotted red line. Scale bars, 200 μm. Middle: Invasion distance of invaded glioma cells from the edge of the bead (shown as percentage of sh*control*). Right: Proliferation of glioma cells normalized to area, representing cellular density.

Ablation of VEGF/VEGFR-2 signaling both genetically and pharmacologically using AZD2171 (100 nM) or BEV (1–3 mg/ml) increased cell invasion (Figure 4A; Figure S8A upper row) without affecting migration (Figure S8A, lower row). As AZD2171 is a multikinase inhibitor, we additionally assessed invasion of LN-308 sh*VEGFR-2* cells as well as VEGFR-2-negative LN-229 cells following treatment with AZD2171. LN-308 sh*VEGFR-2* cells responded to AZD2171 treatment with a strong increase in invasiveness that was significantly higher than the level observed with sh*control* cells treated with AZD2171 (Figure 4B). In contrast, the VEGFR-2-negative cell line LN-229 showed an insignificant increase in invasiveness and no change in migration, which is basically assessed as chemotaxis upon treatment with AZD2171 (Figure S8B), indicating that VEGFR-2 is a major but not the exclusive factor controlling invasion in response to anti-tyrosine-kinase treatment. Consistent with these results, overexpression of VEGFR-2 in LN-229 cells resulted in reduced invasiveness compared with control cells (Figure 4C). Moreover, analysis of invasion using the organotypic brain slice culture system *ex vivo* corroborated these findings by demonstrating markedly increased invasiveness for LN-308 sh*VEGFR-2* cells (Figure 4D, left and middle), that corresponds to the lower cellular density in tumors formed by sh*VEGFR-2* cells (Figure 4D, right).

Of note, expression of the hepatocyte growth factor (HGF) receptor MET in VEGFR-2-positive glioma cells was heterogeneous. U138MG cells expressed high levels of MET on mRNA and protein levels, whereas LN-308 glioma cells demonstrated almost no MET expression (Figure S8C, left). These levels did not change upon knock-down of *VEGFR-2* (Figure S8C, right).

**Figure 5:**
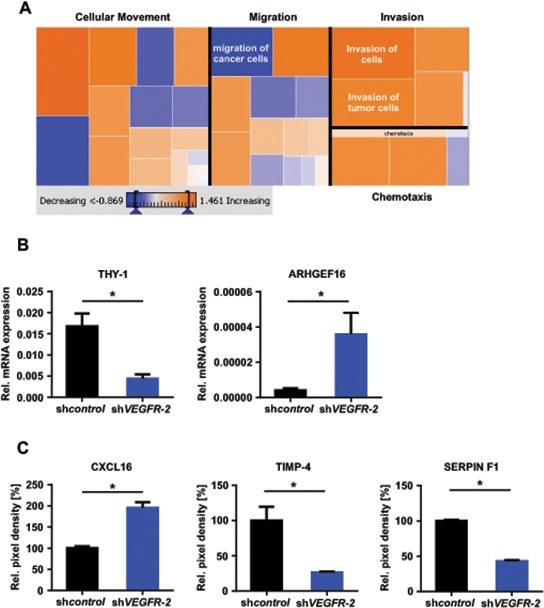
*VEGFR-2* knock-down cells reveal an invasion-prone gene and protein expression profile **(A)** IPA of the LN-308 mRNA microarray. Dysregulated genes known to be involved in “Cellular movement”, “Migration”, “Invasion” and “Chemotaxis” and a predictive regulation was calculated by the expression changes of the genes in each group. Orange fields predict a possible increase of this function by *VEGFR-2* knock-down, blue fields indicate a decrease. **(B)** qRT-PCR analysis, mRNA expression of *THY-1 (CD90)* and *ARHGEF16* in LN-308 sh*control* and sh*VEGFR-2* cells. Each mRNA is displayed relative to *actin* expression. **(C)** Protein levels of CXCL16, TIMP-4 and SERPIN F1 assessed by a proteome profiler angioarray of LN-308 sh*control* and sh*VEGFR-2* cells.

These observations were corroborated by a further IPA analysis of the above-mentioned microarray analysis of LN-308 and U138MG sh*VEGFR-2* cells, focusing on cellular movement, motility, migration and invasion. This analysis revealed that all invasion-relevant topics, especially ‘invasion of cells’ and ‘invasion of tumor cells’, were positively correlated according to the gene expression changes associated with the knock-down of *VEGFR-2*. In contrast, the topics ‘cellular movement’ and ‘migration’ yielded in a less clear prediction of regulation. The topic ‘migration of cancer cells’ was even negatively correlated with the gene expression profile associated with the knock-down of *VEGFR-2* (Figure 5A). This is in line with the findings from our functional *in vitro* assays showing that knock-down of *VEGFR-2* increases invasion but does not affect migration of glioma cells. Finally, we identified and confirmed upregulation of two candidate molecules in response to knock-down of *VEGFR-2* that have been described to exert proinvasive functions: chemokine (C-X-C motif) ligand (CXCL)16 and Rho guanine nucleotide exchange factor (ARHGEF)16 [19, 20]. Correspondingly, THY-1 cell surface antigen (CD90), tissue inhibitor of metalloproteinase (TIMP)-4 and SERPIN F1, previously reported to negatively regulate invasion of cancer cells, were found to be downregulated (Figure 5B, 5C) [21–23].

### Selective knock-down of *VEGFR-2* in glioma cells induces a highly invasive and proangiogenic growth pattern in a xenograft mouse model

**Figure 6:**
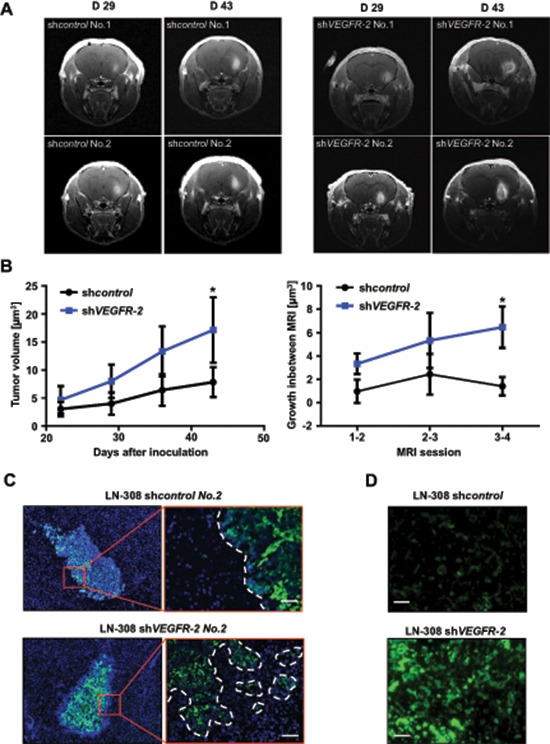
Genetic ablation of *VEGFR-2* in glioma cells produces a more invasive growth phenotype in a xenograft mouse model **(A)** Representative T1 post contrast MRI of two mice harboring LN-308 sh*control* (left) or sh*VEGFR-2* (right) tumors on days 29 and 43 after tumor cell inoculation. **(B)** Left panel: mean tumor volume ± SD assessed in post contrast T1 MRI of mice with LN-308 sh*control* or sh*VEGFR-2* tumors at indicated time points. Right panel: Tumor growth in between two MRI sessions (one week each). **(C)** Immunofluorescent staining for human nestin (green) in brain slides derived from mice in A after termination of the experiment on day 43. Cell nuclei are enhanced with DAPI (blue). Scale bar, 100 μm. **(D)** *In vivo* zymography depicting the activity of MMP-2 and -9 labeled in green. Scale bars, 50 μm.

**Figure 7:**
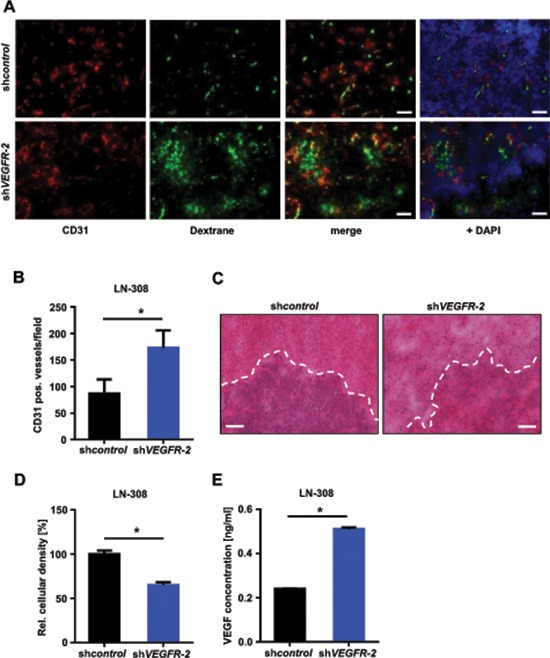
Selective knock-down of glioma cell *VEGFR-2* induces a proangiogenic growth pattern in a xenograft mouse model **(A)** Immunofluorescent images of perfused FITC-dextran (green; vessels), CD31 (red; endothelial cells) and DAPI (blue; cell nuclei). Scale bars, 100 μm. **(B)** Vessel densities in LN-308 sh*control* and sh*VEGFR-2* xenografts. Ten random microscpe fields per tumor were taken at 200-fold magnification and the amount of CD31-positive blood vessels was automatically calculated using ImageJ. **(C)** Representative images of the histological analysis of LN-308 sh*control* and sh*VEGFR-2* xenografts. Hematoxylin/eosin-staining. Scale bars, 200 μm. **(D)** ImageJ-based quantitative assessment of the cellular density in LN-308 sh*control* or sh*VEGFR-2* tumors depicted in Figure 7C. **(E)** Concentrations of VEGF-A were measured by ELISA in the supernatants of LN-308 sh*control* or sh*VEGFR-2* cells after 72 h of incubation.

We evaluated VEGFR-2-specific functions in glioma cells *in vivo* and compared the growth pattern of xenografted LN-308 sh*VEGFR-2* cells with sh*control* cells in CD1 nu/nu mice (*n* = 6 per group). Post contrast T1 magnetic resonance imaging (MRI) was used to segment the tumor and to calculate the tumor volumes. Mice bearing a sh*VEGFR-2* tumor showed larger tumor volumes in all four MRI sessions (Figure 6A, 6B). The mean tumor volumes at day 43 post injection were 7.83 ± 2.67 μm^3^ for sh*control* tumors and 18.1 ± 5.8 μm^3^ for sh*VEGFR-2* tumors (*p* = 0.024). All six sh*control* tumors showed a homogeneous contrast enhancement, whereas five of the six sh*VEGFR-2* tumors appeared inhomogeneous with a hypointense central lesion, diffuse tumor borders and high contrast enhancement, suggesting infiltrative growth and disruption of the blood-brain barrier (Figure 6A). sh*VEGFR-2* tumors histologically displayed a locally destructive growth pattern with marked tumor satellite formation in contrast to sh*control* tumors that all displayed a sharp tumor rim (Figure 6C). This was paralleled by a strong activation of matrix metalloproteinases (MMP)-2 and -9 in sh*VEGFR-2* tumors (Figure 6D) suggesting a proinvasive growth phenotype. Moreover, sh*VEGFR-2* tumors demonstrated a significant increase in CD31-positive blood vessels when compared with control tumors (Figure 7A, 7B) and extravasation of FITC-dextran that had been perfused in a subgroup of mice (Figure 7A) as well as a large peritumoral edema (Figure 7C), indicating leakier vessels and proving a marked disruption of the blood-brain barrier in these tumors. Cellular density was significantly higher in sh*control* tumors when compared with sh*VEGFR-2* tumors, resembling the growth pattern previously observed in *ex vivo* brain slice culture models (Figure 7D). One likely explanation for the proangiogenic reaction is that the VEGFR-2 blockade in glioma cells activates the autocrine/paracrine VEGF/VEGFR-2 signaling pathway by increasing the production of its ligand, VEGF, which could then act on the tumor endothelium. This was supported by the observation that we measured increased VEGF-A secretion into the supernatants of LN-308 sh*VEGFR-2* cells compared with sh*control* cells (Figure 7E).

### Treatment with BEV exerts diminished antiangiogenic but enhanced invasive effects in VEGFR-2-positive tumors

**Figure 8:**
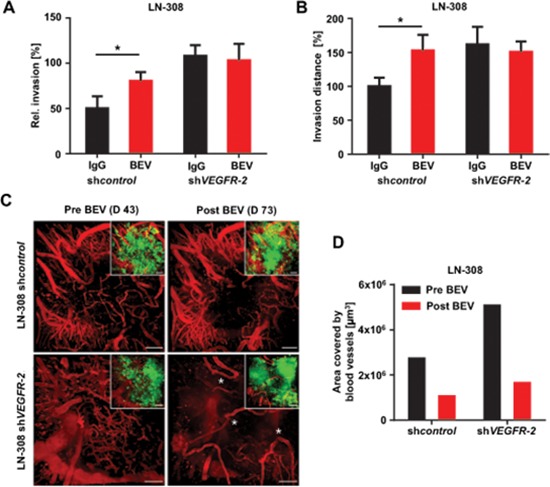
Treatment with BEV exerts diminished antiangiogenic but enhanced invasive effects in VEGFR-2-positive tumors **(A)** RTCA invasion assay of LN-308 sh*control* or sh*VEGFR-2* cells treated with either 1 mg/ml BEV or equivalent IgG. **(B)** Organotypic brain slice culture assays using beads coated with LN-308 sh*control* or sh*VEGFR-2* cells treated with BEV at 3 mg/ml or an equivalent dose of IgG. The distance (normalized to IgG treatment in each line) of invaded glioma cells from the edge of the bead is shown. **(C)** Two-photon microscopic images of the vasculature of the tumor bulk region of LN-308 sh*control* cells (upper panels) or sh*VEGFR-2* cells (lower panels) stereotactically injected into the right mouse cortex before (day 46) and after (day 73) treatment with BEV 15 mg/kg bodyweight every second day. Aberrant loop-forming tumor vessels developing in response to BEV treatment in the sh*VEGFR-2* tumor group are asterisked. The small images show the same region as the corresponding larger images, but illustrate in addition the GFP-fluorescent tumor mass. Blood vessels were stained by intravenous TRITC-dextran injection (vasculature shown in red). Scale bars, 100 μm. **(D)** Quantification of vessel density of two-photon microscopic images of LN-308 sh*control* or sh*VEGFR-2* cells stereotactically injected into the right mouse cortex before (day 46) and after (day 73) treatment with BEV 15 mg/kg bodyweight every second day (see also C).

We then wanted to know, whether BEV-induced invasiveness is restricted to VEGFR-2-expressing glioma cells. We tested BEV treatment in RTCA assays and found that it causes proinvasive effects specifically in VEGFR-2-positive control but not in sh*VEGFR-2* glioma cells (Figure 8A). Of note, BEV-induced invasiveness of VEGFR-2-positive glioma cells was concentration-dependent as treatment with low-dosed BEV (1 mg/ml) resulted in 1.6-fold increased invasion, whereas high-dosed BEV (3 mg/ml) stimulated invasion by more than 3-fold (Figure 4A). Corresponding VEGFR-2-dependent proinvasive effects of BEV were confirmed in the brain slice culture model (Figure 8B). Aiming at integrating the observed cellular effects in one comprehensive animal model, we investigated nude mice with an implanted cranial window upon inoculation of LN-308 sh*control* or sh*VEGFR-2* cells for tumor cell distribution and vessel architecture before and after BEV treatment, using a two-photon microscope. Before treatment, density of tumor-induced blood vessels in sh*VEGFR-2* tumors was significantly higher than in sh*control* tumors (Figure 8C, 8D). Treatment with BEV for 32 days every second day (15 mg/kg bodyweight) only caused a limited antiangiogenic effect in sh*control* tumors (Figure 8C, upper row; Figure 8D). In contrast, the vascular architecture of sh*VEGFR-2* tumors became heavily disrupted upon BEV treatment compared with the pretreatment situation and with control tumors irrespective of BEV treatment (Figure 8C, lower row; Figure 8D). Taken together, these results demonstrate that the presence of VEGFR-2 on glioma cells both hampers antiangiogenesis and predisposes to an adverse evasive response when BEV is applied.

### BEV treatment prolongs survival of patients with PTEN-proficient glioblastomas

**Figure 9:**
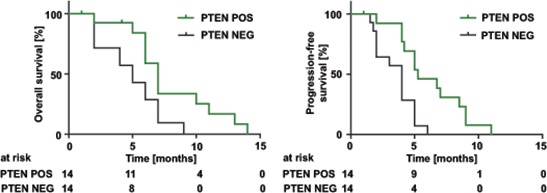
PTEN status predicts overall and progression-free survival in glioblastoma patients treated with BEV Overall survival (left) and progression-free survival (right) of patients with glioblastomas treated with BEV depending on their PTEN status. ‘PTEN NEG’ was defined as: (i) negative PTEN IHC or (ii) positive IHC and an inactivating mutation and/or homozygous deletion in the *PTEN* gene. Positive PTEN IHC without mutation or deletion was evaluated ‘PTEN POS’. Median overall survival: 7 *vs*. 5 months, HR 0.46, 0.13–0.67, (*p* = 0.017). Median progression-free survival 5.2 *vs*. 4 months, HR 0.38, 0.09–0.46, (*p* = 0.002).

Since the introduction of TMZ as first-line therapy for malignant glioma PTEN loss has not been validated as a prognostic biomarker for overall survival [24] as we confirmed in a TCGA database analysis (Figure S9). However, we wanted to know whether PTEN serving as a surrogate marker for glioma cell VEGFR-2 expression is of prognostic value selectively in patients treated with BEV. A clinical case series of twenty-eight patients with recurrent glioblastoma treated with BEV was retrospectively assigned into a PTEN-positive (PTEN POS) or a PTEN-negative (PTEN NEG) group as comprehensively assessed by synoptic immunohistochemical, mutational and CNV analyses. IDH1-mutant tumors were excluded from this analysis because of their overall better prognosis and almost mutual exclusivity with *PTEN* mutations. Patient characteristics at the time of diagnosis were similar in both groups (Table S2). After initiation of BEV therapy, PTEN positivity correlated significantly with prolonged overall survival (median 7 *vs*. 5 months, HR 0.46, 0.13–0.67, *p* = 0.017) and progression-free survival (median 5.25 *vs*. 4 months, HR 0.38, 0.09–0.46, *p* = 0.002) compared with PTEN-negative tumors (Figure 9).

## DISCUSSION

Expression of VEGFR-2 on non-endothelial cells in cancer [14, 25–28] suggests additional functions of the VEGF/VEGFR-2 system in neoplasms besides promoting neovascularization. Specifically, Lu *et al*. recently described a molecular mechanism by which VEGF blockade in glioblastoma cells causes enhanced tumor cell invasion through activation of the HGF receptor MET in a hypoxia-independent manner inducing a program reminiscent of epithelial-to-mesenchymal transition [6]. It is a matter of debate whether evasive resistance is clinically relevant in general or may be restricted to subgroups of patients. It is not clear, whether tumor cell-expressed VEGFR-2 plays a role in this setting [12–14]. Our data clearly suggest the existence of VEGFR-2-expressing tumor cells not only in glioma cell lines and GIC but also in patient-derived glioblastoma tissues, preferentially in tumor infiltration zones.

VEGFR-2-positive glioma cells were exclusively PTEN-deficient suggesting the requirement of PTEN loss and subsequent activation of the PI3K/AKT/mTOR signaling pathway. This was corroborated by our findings that both pharmacological inhibition of mTOR activity using CCI-779 and RNAi-mediated, selective blockade of mTORC1 or 2 signaling each led to a robust downregulation of VEGFR-2. Overexpression of PTEN in PTEN-deficient cell lines reduced VEGFR-2 mRNA and protein expression. However, this reduction was not as strong as through inhibition of mTORC1 or 2, suggesting a more complex regulation of VEGFR-2 *via* the PI3K/AKT/mTOR pathway. Constrictively, we cannot rule out that some of the PTEN-/VEGFR-2-negative tumors express VEGFR-2 in their infiltration zone, since in our series infiltration zones were not available for each tumor specimen.

Moreover, immunohistochemical identification of glioblastoma cell VEGFR-2 positivity is technically demanding and time-consuming, not least because the fraction of VEGFR-2-positive cells within a given tumor is comparatively low. Hence, we propose the PTEN status as assessed by readily available and well-established IHC as a valid surrogate marker that considerably increases the chance of identifying tumors harboring VEGFR-2-positive tumor cells.

We show here that glioma cell VEGFR-2 increases tumor cell proliferation and clonogenicity in different test paradigms, and mediates resistance towards clinically relevant chemotherapy with TMZ, but not irradiation. With a few exemptions, analyses of resistance mechanisms mainly focus on cell-intrinsic mechanisms, which may fall short the magnitude of the problem [29]. While a direct role of VEGFR-2 as a mediator of chemoresistance has not been determined so far, increased sensitivity to alkylating drugs in response to treatment with anti-VEGFR-2 agents is in line with preclinical findings [30, 31]. However, particularly in brain, a combined use of anti-VEGFR-2 compounds and chemotherapy may exert adverse effects, and has not translated into convincing clinical results proving prolonged overall survival [32, 33], largely due to its controversially discussed effects on the tumor vasculature leading to alterations in blood flow, oxygenation status, and delivery of concurrent chemotherapy, i.e., the role of vessel pruning *vs*. normalization.

In our study, glioma cells express VEGFR-2 upon loss of PTEN function through activation of AKT and mTOR. VEGFR-2-dependent effects are paralleled by a molecular expression profile comprising activation of the NF-κB transcription factor complex, several of its downstream genes, and two well-characterized signaling proteins, AKT and NDRG1, that are central to mediate chemoresistance [11, 18]. NF-κB is a transcription factor complex that plays a pivotal role in carcinogenesis and the regulation of immune and inflammatory responses [34]. Activation of the NF-κB complex by the mutant and constitutively active variant vIII of the epidermal growth factor receptor (EGFR) was linked to alkylator resistance in glioma cells [35, 36]. The fact that NF-κB mediates its chemoresistance through inhibition of apoptosis after DNA damage through chemo- or radiotherapy [37] and the presence of an NF-κB binding site within the *O^6^-methylguanine-DNA methyltransferase* (*MGMT*) promoter [38] make an involvement of NF-κB in mediating resistance to TMZ plausible.

We recently reported on the hypoxia-inducible protein NDRG1 as a distinct regulator of resistance to TMZ that does not protect against ionizing irradiation [18]. Here, we found NDRG1 upregulated in VEGFR-2-positive glioma cells, which explains well the increased chemoresistance to TMZ (and not to radiotherapy) observed in these cells. Though otherwise robustly induced with hypoxia in perinecrotic tumor areas of glioblastoma, NDRG1 was found here to be activated in VEGFR-2-positive glioma cells, i.e., in cells that chiefly reside within the tumor infiltration zone where sufficient oxygen supplies are presumably given. Therefore, it seems likely that the VEGFR-2-associated chemoresistance mediated by NDRG1 is hypoxia-independent and alternatively signaled through mTOR.

RNAi-mediated or pharmacological ablation of VEGFR-2 activity increased invasiveness of glioma cells *in vitro* and resulted in a diffuse and locally destructive growth pattern *in vivo*, concomitant with a proinvasive gene expression profile. These effects suggest that VEGFR-2 has a negative impact on glioma cell invasion. The locally destructive growth pattern is reflected by a low cellular density in VEGFR-2-negative tumors suggesting higher tumor volumes on MRI than VEGFR-2-positive tumors. This may be a plausible explanation for our finding that the more proliferative VEGFR-2-positive tumors appear smaller on MRI. We are aware that the increased invasiveness of sh*VEGFR-2* cells is not readily compatible with the localization of VEGFR-2-positive cells to the infiltration zone. Hence, we assume that those tumor cells upregulate VEGFR-2 expression through alternative mechanisms of regulation such as oxygen tensions that are higher in the infiltration zone than in the tumor core, rather than a result of selected less invasive tumor cells. Potential oxygen-dependent effects on glioma cell-specific VEGFR-2 expression will require more detailed investigations in the future.

Previous work described highly invasive and diffuse tumor growth in response to knock-down of *VEGF* resulting in inhibition of the VEGFR-2 pathway [6]. We here complement those findings and identify a similar mechanism by which glioma cell-selective ablation of VEGFR-2 signaling causes a switch to a locally destructive and highly angiogenic growth phenotype that produces abnormal and leaky vessels leading to cerebral edema. The lack of MET expression in one cell line, LN-308, suggests an alternative mechanism besides the previously published pathway through HGF/MET [6]. This is likely to be a result of an induced autocrine/paracrine secretion of the ligand VEGF, which, besides its proangiogenic effects, increases vascular permeability [39], and leads to a more proinvasive phenotype of glioma cells. Induced activity of MMP-2/-9 and expression of ARHGEF16 and CXCL16 together with downregulation of antiinvasive THY-1, TIMP-4 and SERPIN F1 fit well to the locally destructive and invasive growth pattern observed with sh*VEGFR-2* tumors [19–23], and complement the findings reported recently to explain this phenomenon [6]. Given an antiinvasive function of glioma cell VEGFR-2 expression, we consistently found in three independent experimental paradigms *in vitro*, *ex vivo* and *in vivo* that ablation of VEGF/VEGFR-2 signaling, genetically or with BEV treatment, causes proinvasive reactions in the presence of VEGFR-2. Although BEV treatments performed in mouse models are limited in that only human VEGF-A secreted by the xenografted glioma cells is blocked, we found dramatically reduced antiangiogenic effects of BEV in VEGFR-2-positive gliomas, while the absence of VEGFR-2 led to a highly instable vascular system susceptible to abrogation with BEV.

We identified glioma cell VEGFR-2 expression in 19% of all glioblastoma specimens tested. Notably, this percentage roughly corresponds to the recently published portion of recurrent malignant gliomas that displayed distant or diffuse recurrence on MRI scans at the time of failure of BEV-containing treatments [8]. It would have been intriguing to evaluate those gliomas for tumor cell VEGFR-2 positivity, but a lack of sufficient tissue material did not allow such an investigation. Instead, we conducted a retrospective analysis of clinically well-documented patients with recurrent glioblastomas treated with BEV. BEV significantly prolonged both overall and progression-free survival of patients with PTEN-positive tumors when compared with their PTEN-negative counterparts. As this finding supports the view of PTEN as a potential predictor of response to clinically relevant antiangiogenic treatment, these data justify a validation in a prospective large-scale patient cohort.

**Figure 10:**
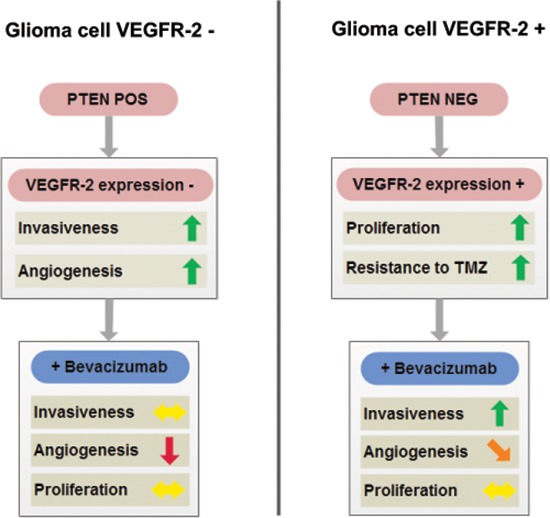
Schematic overview summarizing cellular properties of glioblastomas with (right) or without (left) tumor cell expression of VEGFR-2 and their differential responsiveness to antiangiogenic treatment with BEV Of note, tumor cell expression of VEGFR-2 (right) is met only in a subgroup of PTEN-negative glioblastomas, whereas PTEN-positive tumor cells exclude tumor cell VEGFR-2 positivity (see also Table 1).

Conclusively, we identified two separate subgroups of glioblastomas: (i) PTEN-positive tumors that do not display glioma cell VEGFR-2 positivity (Figure 10, left) and (ii) PTEN-negative tumors with a markedly increased likelihood of glioma cell VEGFR-2 expression (∼40% in our series; Figure 10, right). The first subgroup is less proliferative than the second and is expected to be well-responsive to antiangiogenic treatment, and at a low risk to develop early evasive resistance. The second, in contrast, represents a more aggressive subgroup of glioblastoma being more resistant to chemotherapy with TMZ and at increased risk to develop early diffuse or distant progression upon antiangiogenic therapy with BEV leading to reduced survival. Hence, this latter subgroup is likely to benefit from combined treatment concepts integrating antiangiogenic with antiinvasive mechanisms of action. Alternatively, one could argue not to treat this glioblastoma subgroup with antiangiogenic agents at all. Loss of PTEN can serve as a surrogate marker to identify this subgroup upfront by routine neuropathological methods before antiangiogenic treatment should be considered.

## MATERIALS AND METHODS

### Cell culture, reagents and transfections

The human glioma cell lines LN-18, LN-229, LN-319, T98G, U251, U138MG and LN-428 were purchased from the American Type Culture Collection (Manassas, VA, USA). The human glioma cell line LN-308 was provided by N. de Tribolet. Regular checks for authentity and freedom from infection, e.g. mycoplasms, were done according to the institutional guidelines at the German Cancer Research Center. Details concerning cloning, quantitative reverse transcription PCR analyses (including Table S3), sequencing (including Table S4), shRNA constructs for *VEGFR-2*, siRNA constructs for *Raptor* or *Rictor* and overexpression constructs for *VEGFR-2* or *PTEN* including the respective controls are given in the Supplementary Information.

### Microarray analysis

For gene expression analysis, total RNA was extracted using the RNeasy Kit (Qiagen, Hilden, Germany). RNA integrity was assessed with a 2100 Bioanalyzer (Agilent Technologies, Santa Clara, CA, USA). Only samples with an RNA integrity number (RIN) above seven were used for further processing. The microarray was designed to compare both LN-308 and U138MG sh*VEGFR-2* cells with appropriate controls. Three independent RNA samples from each cell line were used. Analysis of the samples was carried out at the genomics and proteomics core facility of the German Cancer Research Center (Heidelberg, Germany) using an Illumina HT 12 microarray chip. Analysis of corresponding protein networks was done with Ingenuity Pathway Analysis (IPA; Ingenuity Systems, Redwood City, CA, USA). Only proteins regulated in the same direction in both LN-308 and U138MG cells were used for further validation. The microarray data have been deposited in NCBI's Gene Expression Omnibus under accession number GSE61178 (http://www.ncbi.nlm.nih.gov/geo/query/acc.cgi?acc=GSE61178).

### Immunohistochemistry and immunofluorescence

Formalin-fixed paraffin-embedded tissues of human glioblastomas were provided by the Department of Neuropathology, Institute of Pathology, Heidelberg University Hospital, Germany. Sections cut to 3 μm were processed using a Ventana BenchMark XT immunostainer (Ventana Medical Systems, Tucson, AZ, USA). Details are given in the Supplementary Information including an overview of all antibodies used in this work (Tables S5 and S6).

### Clonogenicity

Clonogenic capacity was assessed by seeding 500 glioma cells suspended in culture media and counting of macroscopically visible colonies after 12 days.

### Comparative real-time analysis of cell proliferation and motility and *in vitro* invasion assay

Details are provided in the Supplementary Information.

### Organotypic brain slice culture assay

Details are given in the Supplementary Information.

### Animal experiments

All animal work was approved by the governmental authorities (Karlsruhe, Germany) and in accordance with the NIH ‘Guide for the Care and Use of Laboratory Animals’. Glioma cells were stereotactically implanted into the right brain hemisphere of CD1 nu/nu mice (Charles River Laboratories, Sulzfeld, Germany) at a depth of 3 mm. A detailed description of all animal work including magnetic resonance imaging (MRI) and two-photon microscopy is given in the Supplementary Information.

### Clinical case series

Details are given in the Supplementary Information.

### Statistical analysis

Quantitative *in vitro* data are expressed as mean ± SD, as indicated. All *in vitro* experiments reported here represent at least three independent replications performed in triplicate. Statistical significance was assessed by two-sided Student's t-test (Excel, Microsoft, Seattle, WA, USA). The exact Fisher test was applied to correlate VEGFR-2 with PTEN expression. Univariate survival analyses were performed using the Kaplan-Meier estimator and the log-rank test. Values of *p* < 0.05 were considered significant and asterisked.

## SUPPLEMENTARY MATERIALS AND METHODS FIGURES AND TABLES


