# Supplementary material for: Glioma cell VEGFR-2 confers resistance to chemotherapeutic and antiangiogenic treatments in PTEN-deficient glioblastoma
Source: Oncotarget. 2015 Feb 19;6(31):31050–68. doi: 10.18632/oncotarget.2910 (PMC4741588; doi:10.18632/oncotarget.2910)
Supplement: Supplementary file 1 [file oncotarget-06-31050-s001.pdf]

SUPPLEMENTARY MATERIALS AND METHODS

Cell culture and reagents

Glioma cells were maintained in Dulbecco's Modified Eagle's Medium (DMEM) containing 10% fetal bovine serum (FBS; Perbio; Bonn, Germany), 100 IU/ml penicillin and 100 mg/ml streptomycin (PAA Laboratories, Pasching, Austria).

Tumor specimens were obtained from adult patients diagnosed with glioblastoma after informed consent. Glioma-initiating cell (GIC) cultures were established from freshly dissected tumor tissue. The tumor and neurosphere cultures T269 and T325 were defined and cultured as described [1]. Cells were seeded in neural sphere cell medium (NSCM) containing DMEM:F12 medium enriched with B27 supplement, basic fibroblast growth factor (bFGF) (20 ng/ml), epidermal growth factor (EGF) (20 ng/ml) and leukemia inhibitory factor (LIF) (20 ng/ml) [2]. Human embryonic kidney (HEK) 293T cells were purchased from PromoCell (Heidelberg, Germany) and used for generation of lentiviral particles. Cells were cultured in Iscove's Modified Eagle's Medium (IMEM; PromoCell) containing 10% fetal bovine serum (FBS; Perbio, Bonn, Germany), 100 IU/ml penicillin and 100 mg/ml streptomycin (PAA Laboratories). HeLa cells were used for virus titering and maintained in DMEM containing 10% fetal bovine serum, 100 IU/ml penicillin and 100 mg/ml streptomycin. Human umbilical vein endothelial cells (HUVEC) were purchased from PromoCell (Heidelberg, Germany) and cultured in endothelial cell growth medium (ECGM, PromoCell) with PromoCell supplement mix c-39215, 10% FBS, 100 IU/ml penicillin and 100 mg/ml Streptomycin. HUVEC were only used between passage 3 and 5.

AZD2171 (Cediranib) was kindly provided by Apogenix GmbH (Heidelberg, Germany). CCI-779 (Temsirolimus/Torisel™) was provided by Wyeth Pharma (Muenster, Germany). Temozolomide (TMZ) was provided by Schering-Plough (Kenikworth, NJ, USA). AZD2171, CCI-779 and TMZ were dissolved in dimethyl sulfoxide (DMSO) prior to use in cell culture. The monoclonal anti-VEGF antibody BEV (Avastin™) was purchased from Roche Pharma (Grenzach, Germany), IgG control antibodies (Kiovig™) were purchased from Baxter (Unterschleißheim, Germany), and antibodies were dissolved in 0.9% sodium chloride. Irradiation of cells was performed in a GE Healthcare Buchler (Braunschweig, Germany) radiation device OB58 using a caesium-137 source. Subconfluent glioma cells seeded on small cell culture flasks or 6 cm dishes were irradiated with the indicated dose (usually 1 to 8 Gy) and experiments were performed 48–72 h after irradiation.

RNAi-mediated gene knock-down

shRNA constructs (Sigma-Aldrich, Taufkirchen, Germany) targeting two different regions of the *VEGFR-2* transcript were used:

sh*VEGFR-2_1*:

5′-CCGGCTGGAATGAATACCCTCATATCTCG AGATATGAGGGTATTCATTCCAGTTTTTG-302B9

sh*VEGFR-2_2*:

5′-AATTCAAAAAACCACAGATCATGTGGTTT AAACTCGAGTTTAAACCACATGATCTGTGG-3′.

Non-targeting shRNA (MISSION SHC002, Sigma-Aldrich) was used as a control. shRNA was cloned into a pLKO.1 vector according to standard cloning procedures. Lentiviral particles were produced by co-transfecting psPAX2, pMD2.G and pLKO.1 constructs (TRC1, Sigma-Aldrich) in HEK293T cells using TransIT LT1 (Mirus Bio, Madison, WI, USA). Viruses were harvested by ultracentrifugation 48 h and 96 h after transfection. Infection of LN-308 and U138MG glioma cells with 2 MOI of lentiviral particles was carried out in the presence of 8 mg/ml polybrene (Merck Millipore, Darmstadt, Germany). Medium was changed 24 h after transfection. 48 h later, successfully transfected cells were selected with 2 µg/ml puromycin for at least 10 days. Knock-down efficiency was quantified by real-time polymerase chain reaction (RT-PCR) and immunoblot.

ON-TARGET-plus SMART-pool siRNA by Dharmacon RNA Technologies (Lafayette, CO, USA) was used to knock down Raptor (NM_020761; Smartpool No.L-004107) or Rictor (NM_152756; Smartpool No. L-016984). ON-TARGET-plus siControl Nontargeting Pool (D-001810–10-05, Dharmacon) and transfections without siRNA were used as negative controls.

Cloning and exogenous expression of full-length *VEGFR-2*

For generating the pcDNA3.1-VEGFR-2, a pBS(KS+) vector containing cDNA encoding the N-terminal 2.5 kb of the human *VEGFR-2* coding sequence (pBS(KS+)-VEGFR-2-EZ, EZ for extracellular) was kindly provided by K. Plate (University of Frankfurt, Frankfurt, Germany). As sequencing of the pBS(KS+)-VEGFR-2-EZ construct revealed a mutated start codon, primers were designed to repair this mutation and to enhance translation *via* insertion of a Kozac consensus sequence: the 5′ primer VEGFR-2_1for (5′-ATTAGAATTCATAATGGAGAGCA-3′), containing an *Eco*RI restriction site, a start codon and a Kozac concensus sequence, and the 3′ primer VEGFR-2_1rev (5′-GTCCTGCAAGGATGCATTCTTAA-3′) including a *Nsi*I restriction site were used to amplify cDNA of 1.9

kb encoding the N-terminal end of the VEGFR-2 protein. The purified cDNA product was then restriction digested with *Eco*RI and *Nsi*I and subcloned into the cloning vector pBS(KS+) to generate pBS(KS+)-VEGFR-2-EZ. The missing 1.7 kb of the *VEGFR-2* 3′ end was PCR-amplified from HUVEC cDNA that was kindly provided by H.G. Augustin (German Cancer Research Center, Heidelberg, Germany) using the primers VEGFR-2_2for (5′-CGTCAT GGATCCAGATGAACTCC-3′) and VEGFR-2_2rev (5′-AT ATCTCTAGATTAAACAGGAGG-3′). The amplicon obtained from this PCR and pBS(KS+)-VEGFR-2-EZ (see above) were digested with *Bam*HI and *Xba*I and ligated to generate pBS(KS+)-VEGFR-2 containing cDNA of the full-length *VEGFR-2* open reading frame. For expression in eukaryotic cells, the full-length *VEGFR-2* cDNA sequence was subsequently released from pBS(KS+)-VEGFR-2 using *Kpn*I and *Xba*I and subcloned into the pcDNA3.1(+) expression vector. Successful insertion of the complete *VEGFR-2* coding region was verified by agarose gel electrophoresis and subsequent sequence analysis. LN-229 cells were transfected with either pcDNA3.1-VEGFR-2 or pcDNA3.1 as an empty vector control using Fugene HD (Roche Diagnostics, Mannheim, Germany). Cells overexpressing VEGFR-2 were selected with G418 (600 mg/l) for five days. Overexpression of VEGFR-2 was determined by immunoblot. The GenBank accession numbers for human VEGFR-2 are NM_002253 (mRNA) and NP-002244 (protein).

Exogenous expression of PTEN

A pDONR201 cloning vector containing the full-length human *PTEN* sequence was purchased from the Genomics and Proteomics Core Facility (Germany Cancer Research Center, Heidelberg, Germany). Cloning into the pDEST-FLAGc tagged expression vector was performed using the Gateway™ technology according to standard protocols. LN-308 and U138MG cells were transfected with the PTEN expression vector or an empty vector control using Fugene HD in a ratio of 2:7. Transfections without a vector served as further negative controls. Successfully transfected cells were selected with G418 (800 µg/ml). PTEN overexpression was determined by qRT-PCR and immunoblot. The GenBank accession numbers for human PTEN are NM_000314 (mRNA) and NP_000305 (protein).

Quantitative reverse transcription PCR

qRT-PCR was done as described [3]. All expression levels were normalized to β-actin expression. Primer sequences are given in Table S3.

PTEN Sequencing

DNA was extracted from FFPE material. Extraction was carried out using the automated Maxwell system (Promega, Madison, USA). For PCR, 20 ng of DNA and KOD Hot Start Master Mix (Merck, Darmstadt, Germany) were employed. Briefly, PCR of each exon of *PTEN* was amplified using the specific primers of each exon given in Table S4 and performed in a total reaction volume of 20 µl. PCR was started with an initial polymerase activation step at 95°C for 2 min, followed by 35 cycles beginning with denaturation at 95°C for 20 s, annealing for 45 s at indicated temperatures, and extension at 60°C for 10 s, followed by a final extension at 60°C for 20 min with subsequent cooling to room temperature. The amplification product (2 µl) was submitted to bidirectional sequencing using the BigDye Terminator v3.1 Sequencing Kit (Applied Biosystems, Foster City, USA). Mutations were identified by visual analysis of the sequence chromatograms using Sequence Pilot version 3.1 software (JSI-Medisys, Kippenheim, Germany). Potential biological effects of *PTEN* point mutations were estimated with the mutation assessor (mutationassessor.org, Memorial Sloan Kettering Cancer Center, New York, NY, USA).

PTEN multiplex ligation-dependent probe amplification (MLPA)

Total DNA was obtained from patient specimens as described for *PTEN* sequencing. DNA was processed according to the manufacturer's protocol and the SALSA MLPA probemix P225-D1 PTEN was used (MRC-Holland, Amsterdam, Netherlands). Data analysis was done using the Sequence Pilot 4.1.2 software (JSI-Medisys). A Dosage Quotient (DQ) < 0.7 indicates a heterozygous deletion.

Immunoblot and human angiogenesis array

Preparation of cell lysates and immunoblots were performed as described [4]. Primary and secondary antibodies are given in Tables S5 and S6. For the proteome profiler array, following indicated pretreatment, cells were lysed and 300 µg of protein were used in a proteome profiler human angiogenesis array (R&D Systems, Minneapolis, MN, USA). Pixel density of array spots was quantified using ImageJ (NIH, Bethesda, MD, USA). Mean values of pixel density were compared between control and knock-down after subtraction of background value.

Flow cytometry

Flow cytometry analysis was performed in a BD FACSCanto II flow cytometer (BD Biosciences, Heidelberg, Germany). Fluorescence in a total of 10,000 events per condition was detected and results were analyzed using the FACSDiva 6.1 software (BD Biosciences). *VEGFR-2 flow cytometry*: For extracellular flow cytometry, cells were dissociated with accutase, washed and stained with rabbit polyclonal anti-human VEGFR-2 (1:200, Cell Signaling, Danvers, MA, USA) or a respective isotype control. For intracellular staining, cells were fixed and permeabilized with Cytofix/Cytoperm (BD

Biosciences) prior to staining with the first antibody. As secondary antibody, donkey Alexa 488 anti-rabbit antibody was used (Invitrogen, Karlsruhe, Germany). Specific fluorescence intensity (SFI) was calculated by dividing the mean VEGFR-2 fluorescence signal by the mean isotype signal. *PI flow cytometry*: After treatment as indicated, cells dissociated with trypsin were fixed in ice cold methanol, washed and stained for 1 h in propidium iodide (40 µg/ml) with RNAse (20 µg/ml). Cell cycle distribution was quantified using the Dean-Jett-Fox method.

Determination of VEGF concentration

VEGF concentrations in cell supernatants were quantified using the enzyme-linked immunosorbent assay (ELISA) Quantikine kit (R&D Systems). Following indicated treatment, cell culture supernatants were collected, freed from debris, nonadherent and dead cells by centrifugation (3 min; 4370 × g) and used in the ELISA. The absorbance at 450 nm was determined for each probe using a 96-well plate reader (Thermo Scientific, Karlsruhe, Germany) and used to calculate the respective VEGF concentration (pg/ml) based on a standard curve generated from defined standard samples.

Comparative real-time analysis of cell proliferation and motility

Cell proliferation and invasion were independently monitored using the xCELLigence Real-Time Cell Analyzer system (RTCA; Roche Diagnostics) allowing real-time, label-free cellular analysis. Output of the experiments is a dimensionless cell index, derived as a relative change in measured electrical impedance caused by proliferated or invaded cells, respectively.

Proliferation measurements

Following indicated pretreatment, cells were seeded in quadruplets at a total of 2–5 x 10^3 cells suspended in culture media into each chamber of an RTCA E-plate. Proliferation was monitored for at least 72 h. For experiments measuring sensitivity to TMZ, monitoring was prolonged to 6 days.

Invasion and migration measurements

For invasion experiments, the upper chambers were initially coated with matrigel (1%) and rat tail collagen (4%), but left uncoated for migration experiments. Following indicated pretreatment, glioma cells were seeded in quadruplets at a total of 6 x 10^4 cells suspended in serum free media to each upper chamber of an RTCA CIM-plate. Lower chambers were filled with culture media containing 10% FBS as a chemoattractant. Proliferation and invasion/migration were simultaneously monitored in separate plates of the RTCA system for 24 h and growth curves were analyzed using the RTCA software 1.2. To disentangle invasion/migration-sprecific effects from proliferation-specific effects, the cell index for invasion/migration was normalized to the cell index for proliferation at each monitored time point. A detailed description of the experimental procedure is provided by the manufacturer [5].

In vitro invasion assay

Invasion of glioma cells *in vitro* was assessed in Boyden chamber assays (BD Biosciences) following indicated pretreatments. To disentangle invasion- from proliferation-specific effects, all invasion data presented were normalized to cell viability as assessed by crystal violet staining run in parallel at identical experimental conditions.

Organotypic brain slice culture assay

Preparation of LN-308-coated beads

LN-308 cells were trypsinized and 10^6 cells were mixed with 2000 Cytodex-3 beads (17–0485-01, GE Healthcare) in 1.5 ml of warm culture medium and placed in a FACS tube in the incubator for 4 h, shaking the tube every 20 min. After 4 h, coated beads and medium were transferred to a culture flask and left in the incubator overnight.

Preparation of organotypic slices and grafting of LN-308-coated beads

Organotypic slices were prepared from CD1 mouse pups (postnatal day 7) with a few modifications from previously described methods [6–9]. Briefly, mouse pups were sacrificed by decapitation. Brains were rapidly dissected and placed in cold Gey's balance solution (G9779**,** Sigma-Aldrich). Subsequently, brains were embedded in 5% low melting agarose and cut coronally into 300 µm thick slices using a vibratome. Organotypic slices were placed on millicells (Merck Millipore) in 6-well plates and 1 ml/well of the following culture medium: DMEM containing 25% horse serum (Gibco Life Technologies, Karlsruhe, Germany), 25% Hanks balanced salt solution (Gibco Life Technologies), 25 mM Hepes (Roth, Karlsruhe, Germany), 2 mM $NaHCO_3$ (Sigma-Aldrich), 6.5 mg/ml glucose (Sigma-Aldrich), 2 mM glutamine (Gibco Life Technologies) and 100 U/ml penicillin/streptomycin (Sigma-Aldrich). Subsequently, beads coated with LN-308 sh*control* or sh*VEGFR-2* cells were placed in a sterile petri dish and single beads were picked using a glass capillary and a mouth pipetor under a stereomicroscope. Using the glass capillary and the mouth pipetor, beads were grafted in subcortical areas. For reasons of comparison one bead was grafted per slice, and all beads were implanted in the same subcortical region. Slices with grafted beads were cultured at the interface between air and the culture medium at 37°C and 5% CO_2, and culture medium was changed every 2–3 days (Figure S8).

Treatment of organotypic slices

All slices were treated either with IgG control or BEV at 3 mg/ml during 5 days. Medium containing IgG or BEV was replaced every 2–3 days. At the end of the treatment, slices were fixed in 4% PFA containing 4% sucrose overnight at 4°C and processed for immunostaining, imaging and analysis.

Immunostaining and imaging

Fixed slices were counterstained with the nuclear marker TOPRO-3 (T3605, Invitrogen) for 30 min in PBS at room temperature and subsequently slices were mounted on microscope slides for imaging. Control slices were immunostained for different cellular markers to test viability of the cultures. Briefly, slices were blocked in PBS containing 0.3% Triton X-100, 1% BSA (Roth) and 2% normal donkey serum (Dianova) for 2 h at room temperature and then incubated with antibodies for the neuronal marker NeuN (AB5320, Merck Millipore) and the glia marker GFAP (Z0334, Dako, Glostrup, Denmark) in blocking buffer overnight at 4°C. Next day, slices were washed in PBS containing 0.3% Triton X-100 and incubated with Alexa-labeled secondary antibodies. Slices were mounted on microscope slides for subsequent imaging. Imaging was performed using a Zeiss LSM 510 META confocal microscope (Zeiss, Jena, Germany).

Analysis of cell proliferation and invasion

Image analysis was performed using ImageJ (NIH, Bethesda, MD, USA). Proliferation was calculated according to the fluorescence intensity values of LN-308 sh*control* or sh*VEGFR-2* GFP-positive cells in the maximum projection image. Invasion was quantified by measuring the area covered by LN-308 sh*control* or sh*VEGFR-2* GFP-positive cells within the slice and by measuring the distance (invasion distance) from the border of the bead to the invasion front of LN-308 sh*control* or sh*VEGFR-2* GFP-positive cells within the slice. Only beads for which initial invasion of cells was observed were considered for the analysis.

Immunohistochemistry and immunofluorescence

Staining procedure included a pretreatment with cell conditioner 1 for 60 min. Pretreatment was followed by incubation with rabbit anti-human VEGFR-2 antibody (55B11, monoclonal antibody, Cell Signaling) or rabbit anti-human PTEN (Cell Signaling) at 37°C for 30 min. Incubation was followed by Ventana standard signal amplification, UltraWash, counter-staining with one drop of hematoxylin for 4 min and one drop of bluing reagent for 4 min. For visualization, the ultraView™ Universal DAB Detection Kit (Ventana Medical Systems, Tucson, AZ, USA) was used. For immunofluorescent labeling of human tumor tissues, sections underwent identical pretreatment and were incubated with goat anti-human VEGFR-2 (R&D Systems) and either mouse anti-human IDH1R132H (Dianova, Hamburg, Germany), or mouse anti-human GFAP (Dako), respectively at 4°C over night. Slides were afterwards incubated with secondary antibodies (Alexa Fluor® 488 nm and 568 nm; both Invitrogen) for 30 min at room temperature and covered with mounting medium containing 4′,6-diamidino-2-phenylindole (DAPI; Vector Laboratories, Burlingame, CA, USA). For immunofluorescent analysis of mouse brains, sections (5 µm) were treated as follows: *Nestin Staining*: Sections were fixed with ice cold methanol before staining with the primary antibody rabbit-anti human nestin (1:200, Millipore, Billerica, MA, USA) for 2.5 h at room temperature. Unspecific binding was ruled out by staining proximate slices with a rabbit isotype antibody (1:1000, Southern Biotech, Birmingham, AL, USA). Sections were incubated with a FITC-conjugated anti-rabbit antibody (1:200, Invitrogen) for 20 min before mounting with Vectashield DAPI mounting medium (Vector Laboratories). *Dextran Staining*: Animals were perfused with FITC-labeled dextran immediately before sacrification by injection into the tail vein. Brain sections were fixed in ice cold methanol for 10 min and mounted with DAPI-containing mounting medium. *CD31 Staining*: Blood vessel stainings were conducted with a rat anti-mouse CD31 antibody (BD Biosciences) followed by Alexa Fluor® 546 goat anti-rat IgG secondary antibody (Invitrogen) and DAPI counter-staining. An overview of all primary and secondary antibodies used in this work is given in Tables S5 and S6, respectively. At least ten microscopic images (Cell Observer, Zeiss) per tumor were taken and fluorescent CD31-positive vessels were automatically counted by the NIH ImageJ software.

In situ zymography

Mapping of matrix metalloproteinase (MMP) activity was achieved by *in situ* zymography as described before [10]. Briefly, brain sections were incubated with 40 µg/ml Oregon Green 488-conjugated gelatin (Molecular Probes, Eugene, OR, USA). Addition of EDTA at 10 mmol/l served as a negative control. Sections were counterstained with DAPI and green fluorescent gelatinase activity was visualized (Cell Observer, Zeiss).

Animals, cranial window implantation and tumor cell injection

Cranial window implantation in 8–10 week old male NMRI nu/nu nude mice (Charles River) was performed as described [11]. Briefly, mice were first anesthetized with ketamine/xylazine (100 mg/kg ketamine, 10 mg/kg xylazine) intraperitoneally. After removal of the skin, a round cranial window (6 mm diameter) was drilled and

the dura mater was removed carefully. After application of physiologic sodium chloride solution, the brain was covered by a round cover glass and sealed with dental acrylic glue. After the window was implanted, we waited two to three weeks until tumor implantation to let the mice recover from the first procedure, to reduce the duration of the first operation and to allow the brain to adapt to the cranial window. For tumor injection, mice were again anesthetized, the cover glass was transiently removed and 4 x 10^4 LN-308 sh*control* (*n* = 5 mice) or LN-308 sh*VEGFR-2* (*n* = 5 mice) glioma cells were stereotactically injected into the right mouse cortex of each mouse using a Hamilton microsyringe. Postoperatively, mice received analgetic (carprophen), antibiotic (moxifloxacin) and antiedematous/antiinflammatory (dexamethasone, single dose after cranial window implantation only) treatment. BEV was used to inhibit tumor cell-derived human VEGF-A and administered intraperitoneally at doses of 15 mg/kg bodyweight every second day from day 46 after tumor cell injection.

Magnetic resonance imaging

All scans were performed on a 9.4 T horizontal bore MR scanner (BioSpec 94/20 USR, Bruker BioSpin GmbH, Ettlingen, Germany) with a four channel phased array surface coil. The animals were under inhalation anaesthesia with 1.5–2.5% isoflurane vaporized in compressed air (flow: 1.2 l/min). Prior to image acquisition, the animal received 100 µl of Omniscan (0.5 mmol/ml, GE Healthcare Buchler GmbH, Germany) contrast agent injected i.p. for tumor enhancement. A T1-weighted Rapid Acquisition with Refocused Echoes (RARE) sequence (TR/TE: 905/8.5 ms, NA: 3, echo train length: 2, matrix: 256 x 256, pixel spacing: 0.078 x 0.078 mm, slice thickness: 0.3 mm with an inter-slice spacing of 0.3 mm, number of slices: 25, total time of 5 min 48 s) was acquired for volumetric monitoring. Volumetric analysis was undertaken in Amira® 5.4 (Visage Imaging GmbH, Berlin, Germany) with a semi-automated segmentation procedure of the contrast-enhanced tumor for each measurement session.

Two-photon microscopy

A Chameleon Ultra II laser (690–1040 nm, Coherent) linked to a two-photon microscope (LSM 7 MP; Zeiss) was used for *in vivo* multiphoton laser scanning microscopy. After the tumor had formed (approximately 30 days post injection), mice were imaged once a week under a volatile anesthesia with 1% isoflurane in O_2. For a constant body temperature, mice were placed on a heating pad and a rectal thermometer controlled the heating frequency. Laser power was tuned as low as possible in order to reduce phototoxicity, tissue damage and interference with tumor and host cells. TRITC was injected into the tail vein at each time point (5 mg/mL, 2 mol/L Da MW; Invitrogen) to label the vasculature. Images were acquired at a wavelength of 850 nm. To reduce the duration of the imaging procedure, two representative regions (infiltration zone and main tumor) per mouse were chosen (as a z-stack, 600 µm x 600 µm x 400 µm) and followed over time. Pictures were processed using Imaris software (Bitplane, Zurich, Switzerland).

TCGA data analysis

To assess the influence of PTEN alterations on VEGFR-2 expression, mutation, copy number variation (CNV) and data from reverse phase protein arrays (RPPA) as well as clinical survival data were obtained from the database of The Cancer Genome Atlas (TCGA, http://cancergenome.nih.gov) and from cBioPortal [12] at August 4, 2014. Patient specimens (*n* = 139) with available mutation and CNV data for *PTEN* as well as RPPA data for VEGFR-2 and CD31 were divided into two groups: (i) a PTEN-negative (NEG) group as defined by the presence of mutation and/or a homozygous loss of *PTEN*; (ii) a PTEN-positive (POS) group without mutation or homozygous loss of *PTEN*. RPPA data for VEGFR-2 and CD31 were analyzed. Clinical survival data were compared for patients (*n* = 254) with available *PTEN* mutation and CNV data and analyzed using the Kaplan-Meier estimator and the log-rank test.

Clinical case series

The German law allows individual treatments of patients in an otherwise desperate medical situation. The 28 patient cases summarized were treated with Bevacizumab on an individual basis according to high institutional standards at the University Medical Center in Heidelberg, Germany, for example, biweekly clinical assessment and 6-weekly MRI, but not within a clinical trial protocol.

The PTEN status was defined as follows:

PTEN negative (NEG)

1. Tumors without tumoral expression of PTEN as assessed by immunohistochemistry (IHC).
2. Tumors with positive PTEN IHC and mutations in the *PTEN* gene that were either assessed as having high impact on PTEN function using the mutation assessor (http://mutationassessor.org, Memorial Sloan Kettering Cancer Center, New York, NY, USA), or being either nonsense or frame-shift mutations, or deletions.

PTEN positive (POS)

Tumors with tumoral PTEN expression as assessed by IHC and no mutation in the *PTEN* gene or mutations that were assessed having low or medium impact on PTEN function using the mutation assessor.

Tumor response was assessed according to the Response Assessment in Neuro-Oncology Working Group criteria [13]. Overall survival (OS) was calculated from the first treatment with BEV until death. Progression-free survival was determined from the first treatment with BEV until clinical or radiographic tumor progression, depending on what occurred first. By the time of data analysis, complete follow-up information was available for all patients included in the study.

REFERENCES

1. Rieger J, Lemke D, Maurer G, Weiler M, Frank B, Tabatabai G, Weller M, Wick W. Enzastaurin-induced apoptosis in glioma cells is caspase-dependent and inhibited by BCL-XL. J Neurochem. 2008; 106:2436–2448.
2. Hemmati HD, Nakano I, Lazareff JA, Masterman-Smith M, Geschwind DH, Bronner-Fraser M, Kornblum HI. Cancerous stem cells can arise from pediatric brain tumors. Proc Natl Acad Sci U S A. 2003; 100:15178–15183.
3. Opitz CA, Litzenburger UM, Lutz C, Lanz TV, Tritschler I, Koppel A, Tolosa E, Hoberg M, Anderl J, Aicher WK, Weller M, Wick W, Platten M. Toll-like receptor engagement enhances the immunosuppressive properties of human bone marrow-derived mesenchymal stem cells by inducing indoleamine-2,3-dioxygenase-1 via interferon-beta and protein kinase R. Stem Cells. 2009; 27:909–919.
4. Hertenstein A, Schumacher T, Litzenburger U, Opitz CA, Falk CS, Serafini T, Wick W, Platten M. Suppression of human CD4+ T cell activation by 3,4-dimethoxycinnamonyl-anthranilic acid (tranilast) is mediated by CXCL9 and CXCL10. Biochem Pharmacol. 2011; 82:632–641.
5. Bird CKS. Real-time, label-free monitoring of cellular invasion and migration with the xCELLigence system. Nature Methods. 2009.
6. Stoppini L, Buchs PA, Muller D. A simple method for organotypic cultures of nervous tissue. J Neurosci Methods. 1991; 37:173–182.
7. Ohnishi T, Matsumura H, Izumoto S, Hiraga S, Hayakawa T. A novel model of glioma cell invasion using organotypic brain slice culture. Cancer Res. 1998; 58:2935–2940.
8. Aaberg-Jessen C, Norregaard A, Christensen K, Pedersen CB, Andersen C, Kristensen BW. Invasion of primary glioma- and cell line-derived spheroids implanted into corticostriatal slice cultures. Int J Clin Exp Pathol. 2013; 6:546–560.
9. Almodovar S, Cicalini S, Petrosillo N, Flores SC. Pulmonary hypertension associated with HIV infection: pulmonary vascular disease: the global perspective. Chest. 2010; 137:6S–12S.
10. Weiler M, Bahr O, Hohlweg U, Naumann U, Rieger J, Huang H, Tabatabai G, Krell HW, Ohgaki H, Weller M, Wick W. BCL-xL: time-dependent dissociation between modulation of apoptosis and invasiveness in human malignant glioma cells. Cell Death Differ. 2006; 13:1156–1169.
11. von Baumgarten L, Brucker D, Tirniceru A, Kienast Y, Grau S, Burgold S, Herms J, Winkler F. Bevacizumab has differential and dose-dependent effects on glioma blood vessels and tumor cells. Clin Cancer Res. 2011; 17:6192–6205.
12. Cerami E, Gao J, Dogrusoz U, Gross BE, Sumer SO, Aksoy BA, Jacobsen A, Byrne CJ, Heuer ML, Larsson E, Antipin Y, Reva B, Goldberg AP, *et al*. The cBio cancer genomics portal: an open platform for exploring multidimensional cancer genomics data. Cancer Discov. 2012; 2:401–404.
13. Wen PY, Macdonald DR, Reardon DA, Cloughesy TF, Sorensen AG, Galanis E, Degroot J, Wick W, Gilbert MR, Lassman AB, Tsien C, Mikkelsen T, Wong ET, *et al*. Updated response assessment criteria for high-grade gliomas: response assessment in neuro-oncology working group. J Clin Oncol. 2010; 28:1963–1972.

SUPPLEMENTARY FIGURES AND TABLES

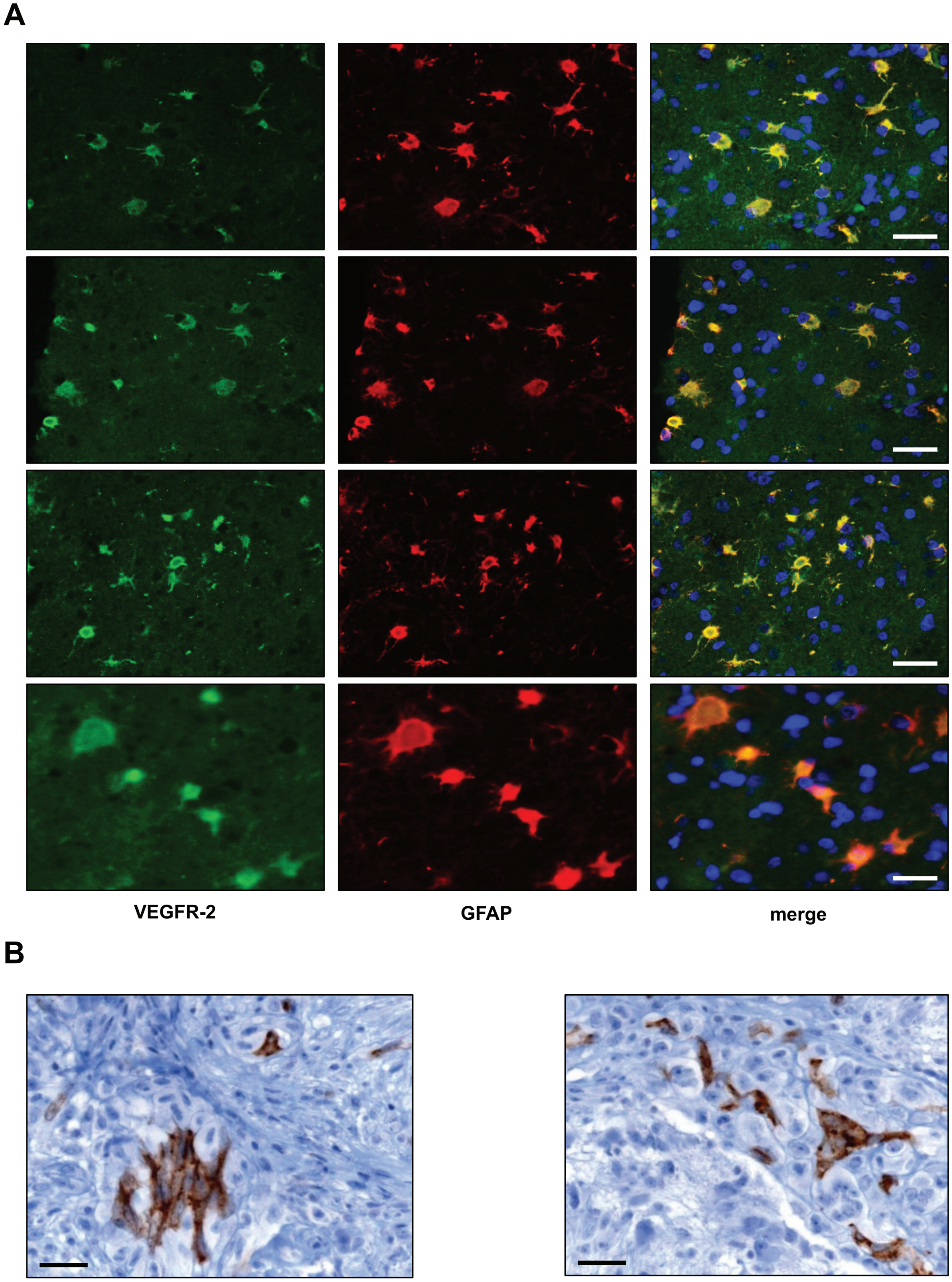

Supplementary Figure S1: Glioma cell VEGFR-2 expression in glioblastoma tissues. (A) VEGFR-2 expression of glial fibrillary acidic protein (GFAP)-positive glioblastoma cells within the tumor infiltration zone. Scale bars in upper three rows, 40 µm; scale bar in lowest row, 20 µm. **(B)** VEGFR-2-specific IHC in glioblastoma illustrating VEGFR-2-positive clusters of tumor cells not forming a vascular lumen. Scale bar, 25 µm.

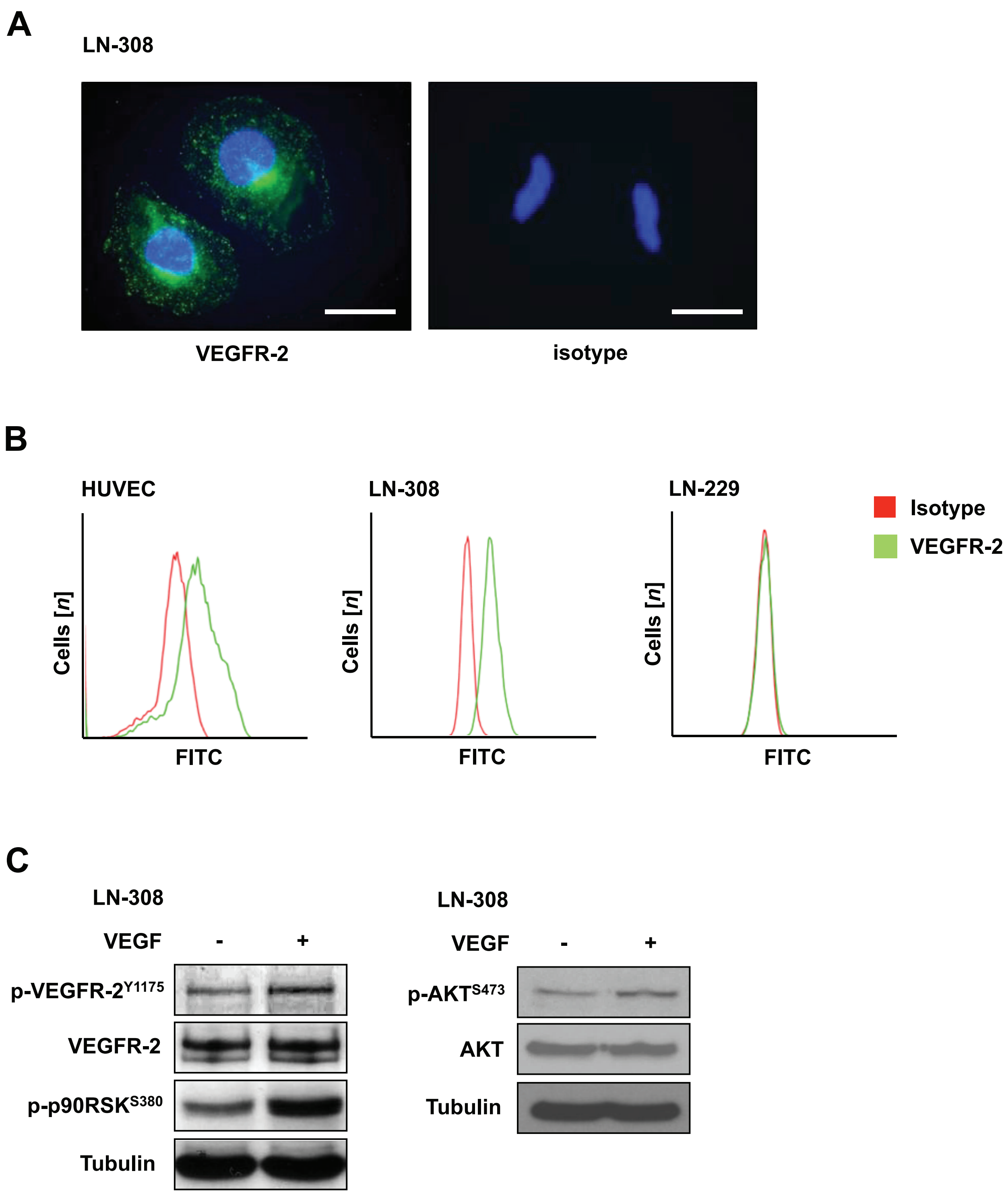

Supplementary Figure S2: VEGFR-2 expression and signaling. **(A)** VEGFR-2-specific immunoflourescence in LN-308 glioma cells. VEGFR-2 is shown in green, DAPI in blue. Scale bars, 10 µm. **(B)** Flow cytometry of HUVEC, LN-308 and LN-229 cells. Fluorescence of cells stained with anti-VEGFR-2 antibody is shown relative to isotype control. **(C)** Immunoblot analysis of LN-308 glioma cells following stimulation with VEGF (50 ng/ml) for 5 minutes.

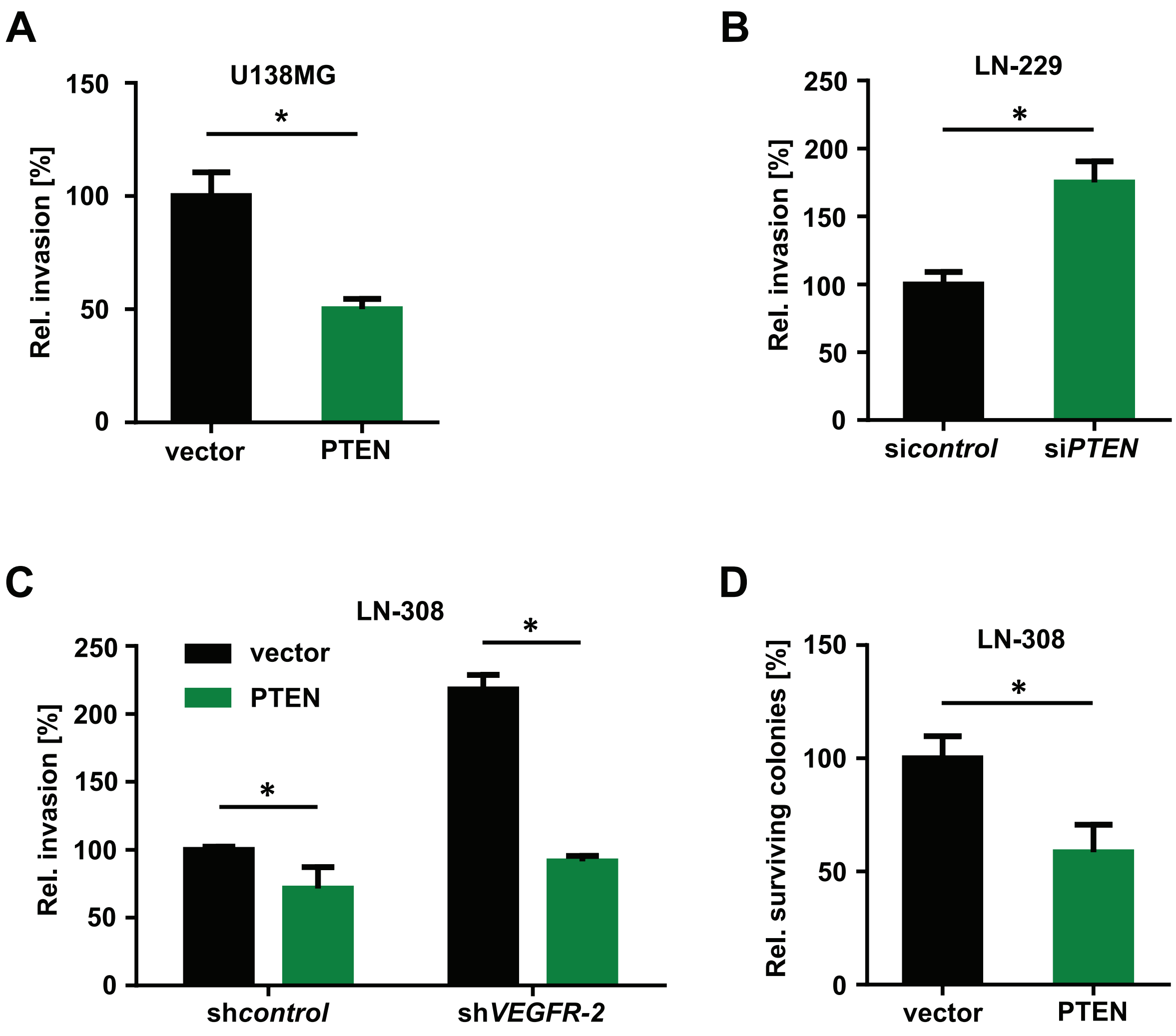

Supplementary Figure S3: Effects of PTEN on invasion and clonogenicity (A) Matrigel invasion assay with U138MG glioma cells transfected with empty vector control or *PTEN*. **(B)** Matrigel invasion assay with LN-229 glioma cells transfected with control siRNA or si*PTEN*. **(C)** Matrigel invasion assay with LN-308 sh*control* and sh*VEGFR-2* cells additionally transfected with either empty vector control or *PTEN*. **(D)** Clonogenicity assay of U138MG glioma cells transfected with empty vector control or *PTEN*.

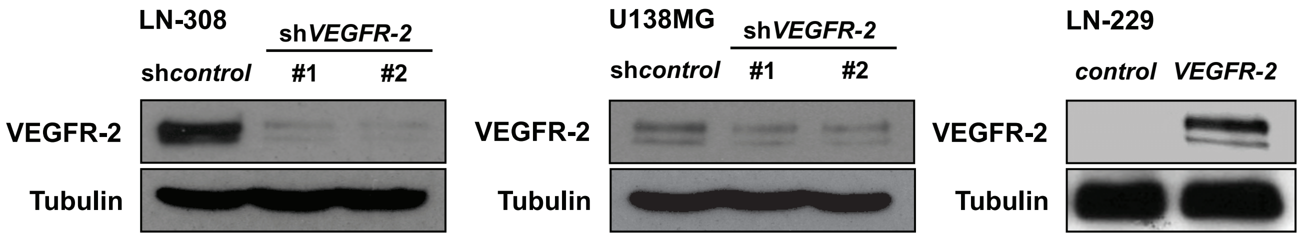

Supplementary Figure S4: Immunoblot analysis of control (sh*control*) or stable *VEGFR-2* knock-down (sh*VEGFR-2*) transfectants of LN-308 (left) and U138MG (middle) glioma cells. shRNAs targeting two different regions of *VEGFR-2* are shown. Immunoblot analysis of stably VEGFR-2-overexpressing LN-229 glioma cells (right). Tubulin served as a loading control.

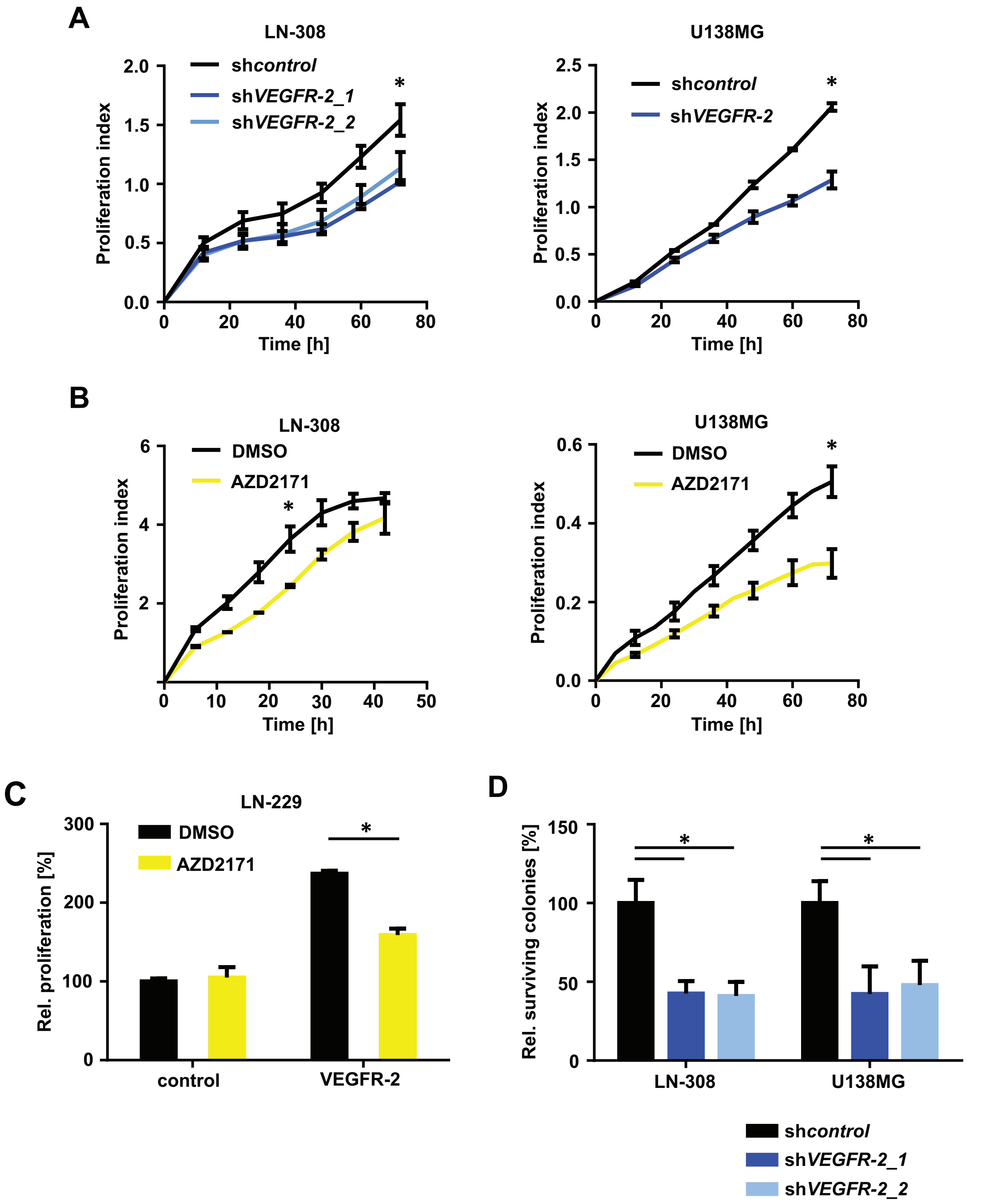

Supplementary Figure S5: VEGFR-2 activity increases proliferation of glioma cells. **(A)** RTCA proliferation assays of LN-308 (left) and U138MG cells (right). 2,500 cells were seeded into each well. **(B)** RTCA proliferation assay with LN-308 cells (left) and U138MG cells (right) treated with AZD2171 (100 nM) or DMSO (diluent). **(C)** RTCA proliferation assay with VEGFR-2-overexpressing LN-229 cells compared with control cells following pretreatment with either DMSO or AZD2171 (100 nM) for 72 h. **(D)** Clonogenic survival of U138MG sh*control* or sh*VEGFR-2* glioma cells. The surviving fraction was calculated on day 12 after seeding.

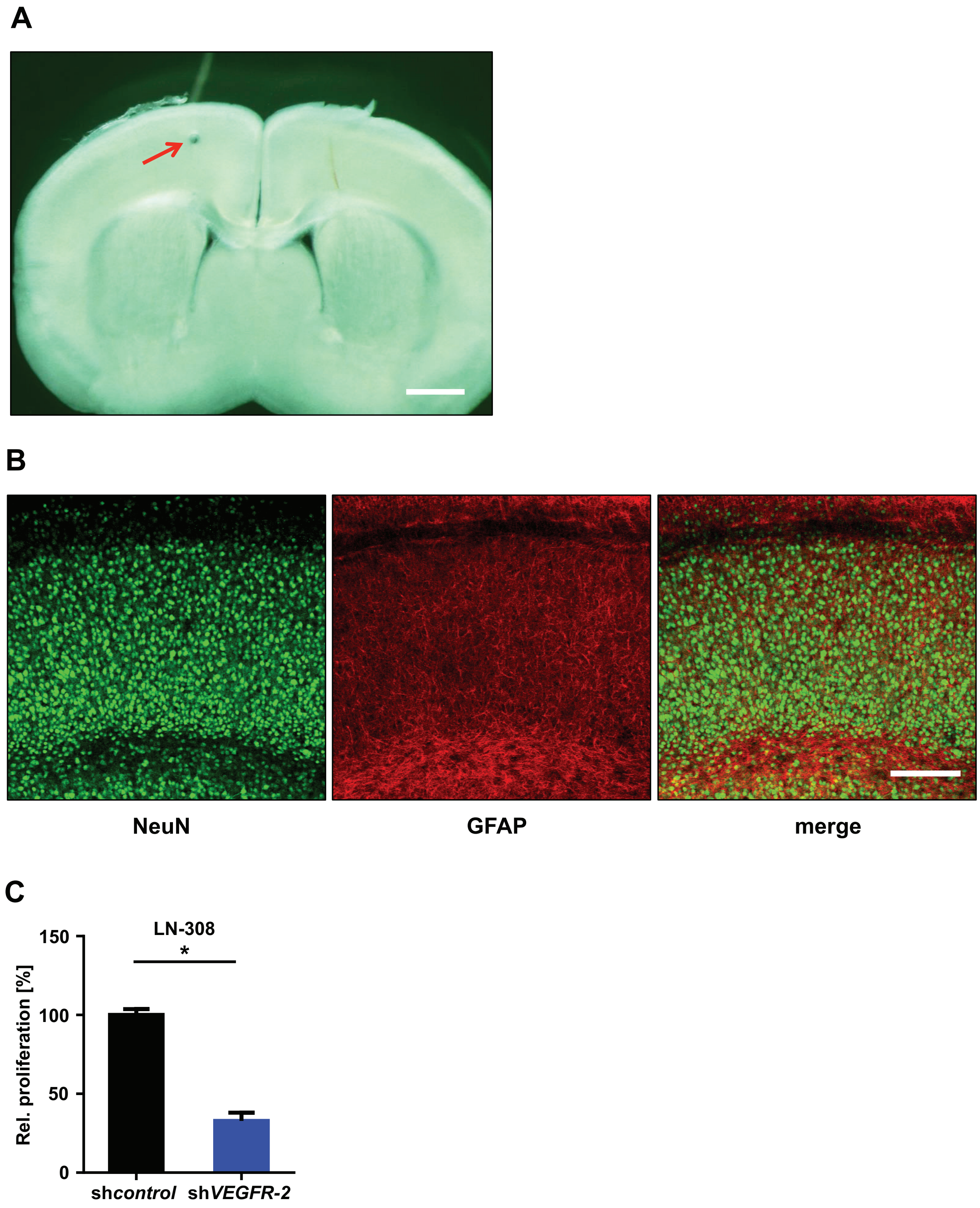

Supplementary Figure S6: Experimental setup of the brain slice culture experiments. (A) Overview of the experimental set up of an *ex vivo* organotypic brain slice culture showing a brain slice (thickness 300 µm) of a CD1 mouse pup (postnatal day 7) placed in a 6-well plate. The arrow marks a bead coated with LN-308 glioma cells after engraftment in the subcortical brain region. Scale bar, 1 mm. **(B)** Immunostainings of brain slices after 7 days in culture showing normal expression and distribution of neurons (NeuN) and glia cells (GFAP). Scale bar, 200 µm. **(C)** Organotypic brain slice culture assay with grafted LN-308 sh*control* or sh*VEGFR-2* glioma cells coated on cytodex beads. The mean fluorescence intensity was used as a measurement for proliferation (both cell lines express similar levels of GFP).

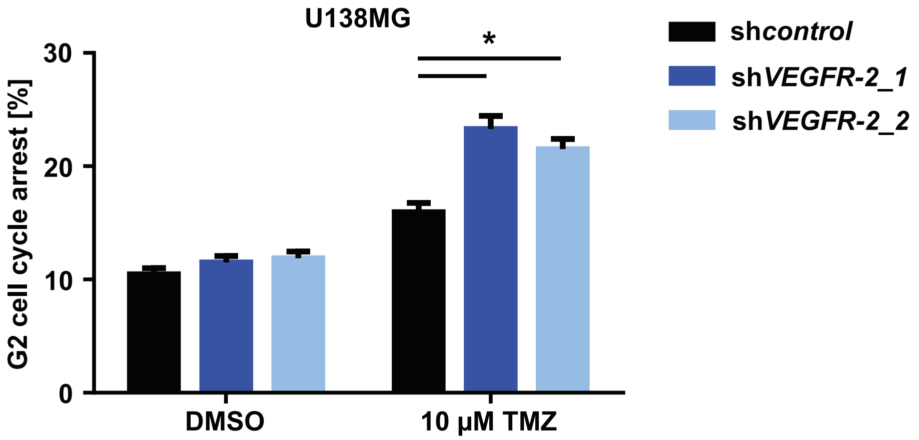

Supplementary Figure S7: Flow cytometry after propidium iodide staining of U138MG sh*control* and sh*VEGFR-2* cells treated with DMSO or TMZ (10 µM) for 72 h. G2-arrested cells were compared with all cells in each respective sample.

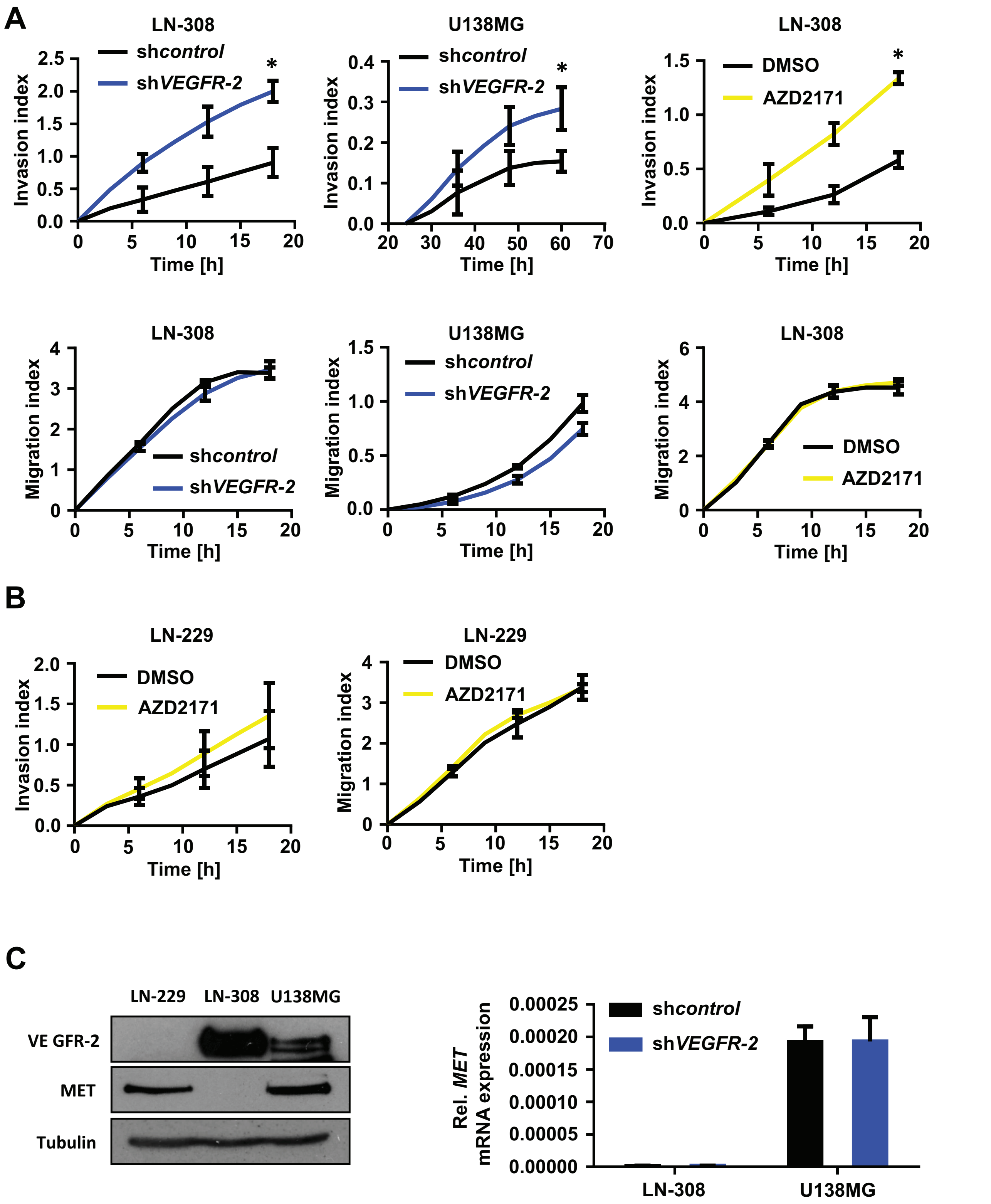

Supplementary Figure S8: **(A)** RTCA invasion assays (upper row) and RTCA migration assays (lower row) of LN-308 and U138MG sh*control* and sh*VEGFR-2* glioma cells as well as LN-308 cells after treatment with DMSO or AZD2171 (100 nM). **(B)** RTCA invasion assay (left) and RTCA migration assay (right) of LN-229 glioma cells following treatment with AZD2171 (100 nM) for 72 h. **(C)** Left, immunoblot analysis of VEGFR-2 and MET in LN-229, LN-308 and U138MG glioma cells. Tubulin served as a loading control. Right, *MET* mRNA expression levels of LN-308 and U138MG glioma cells harboring sh*control* or sh*VEGFR-2* constructs.

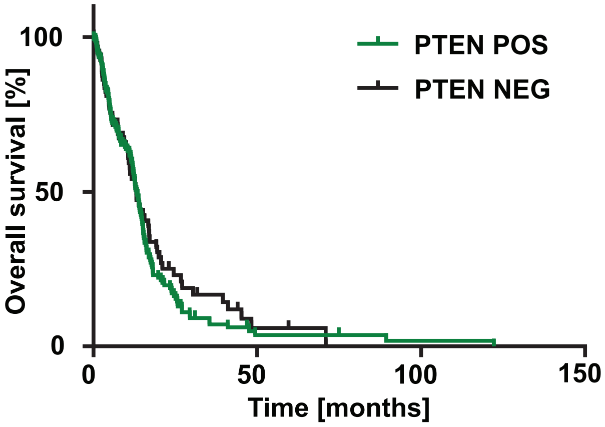

Supplementary Figure S9: PTEN status is not predictive for survival in glioblastoma patients of the TCGA database. OS of patients of the TCGA database depending on the PTEN status. The ‘PTEN NEG’ group was defined as specimens with mutation and/or homozygous PTEN loss. The ‘PTEN POS’ group was defined as specimens without mutation or homozygous loss of PTEN.

Supplementary Table S1: VEGFR-2 expression and PTEN status in glioma cell lines and GICs.VEGFR-2 mRNA and protein expression data were obtained by qRT-PCR and immunoblot analyses (see also Fig. 1). The PTEN status was assessed on grounds of recently published data based on mutational, promoter methylation and expression analyses for the same cells.

	VEGFR-2			PTEN
Glioma cells	mRNA	Protein	Status	Status
LN-308[a]	++	++	POS	NEG
U138MG[a]	+	+	POS	NEG
LN-229[a]	-	-	NEG	POS
LN-428[a]	-	-	NEG	POS
LN-319[a]	(+)	-	NEG	POS
LN-18[a]	-	-	NEG	POS
T98G[a]	-	-	NEG	POS
U251[a]	(+)	-	NEG	POS
T325[b]	++	+	POS	NEG
T269[b]	-	-	NEG	POS

[a]Human glioma cell line.
[b]human GIC culture.
Abbreviations: -, no expression; (+), weak expression; +, moderate expression; ++, strong expression; POS, positive; NEG, negative.

Supplementary Table S2: Baseline characteristics of tumor specimens and corresponding patients subjected to survival analysis (cf. Figure 6E).

	PTEN POS	PTEN NEG	*p*
Total [*n*]	14	14	
**Tumor characteristics**:			
VEGFR-2 POS [*n*], (%)	0 (0)	5 (36)	0.041
VEGFR-2 NEG [*n*], (%)	14 (100)	9 (64)	
IDH1 mut [*n*], (%)	0 (0)	0 (0)	
**Patient characteristics**:			
Mean age [years], (range)	49.5 (33–69)	52.5 (34–72)	0.454
Female/male [%]	3/11	4/10	1.000
Median PFS [months]	5.25	4.00	0.002
Median OS [months]	7.00	5.00	0.017

Abbreviations: POS, positive; NEG, negative.

Supplementary Table S3: qRT-PCR primers

Target	Forward primer (5′-3′)	Reverse primer (5′-3′)
ARHGEF16	CCTGATGGATACGCTCTG	GGACTTGACCTTGCTGAA
CD133	CATCCACAGATGCTCCTAAGGC	AAGAGAATGCCAATGGGTCCA
CXCL16	GACATGCTTACTCGGGGATTG	CAGTGATCCTACTGGGAGGGT
Laminin A5	CACCCTCTCGACTCGCCCCA	GCCGGCAGTAGCATCGACCC
PTEN	TAAAGGCACAAGAGGCCCTA	AGGTAACGGCTGAGGGAACT
Raptor	TAGTTTTGTGCCTGAATGTTGGT	GAGCTTTCTGAGGACCCATCG
Rictor	CGAGTACGAGGGCGGAATG	TCAGATGGCCTAGCTTTCTCATA
THY-1 (CD90)	CTTGCAGGTCTCCCGAGGGC	GCGGCAGTCCAGACGAAGGC
VEGFR-2	GCGTGGTCAGGCAGCTCACA	GGGGATTCCCAGATGCCGTGC

Supplementary Table S4: *PTEN* sequencing primers

Target	Forward primer 5′-3′	Reverse primer 5′-3′
Exon 1	GCAGCTTCTGCCATCTCTCT	CAGCCGCAGAAATGGATAC
Exon 2	GTTTGATTGCTGCATATTTCA	TCTAAATGAAAACACAACATGAA
Exon 3	ATAGAAGGGGTATTTGTTGGA	CCTCACTCTAACAAGCAGATA
Exon 4	ATTCAGGCAATGTTTGTTAG	CAACATAGTACAGTACATTC
Exon 5	TTCTGAGGTTATCTTTTTACCACA	GGAAGAGGAAAGGAAAAACATC
Exon 6	CTTCTCTTTTTTTCTGTCC	AAGGATGAGAATTTCAAGCA
Exon 7	AAAGGCATTTCCTGTGAAATAA	TTGGATATTTCTCCCAATGAAAG
Exon 8	CATAGGTGACAGATTTTCTTTTT	CAGCTGTACTCCTAGAATTAAACA
Exon 9	TGGGTTTTCATTTTAAATTTTCTTTC	CATGGTGTTTTATCCCTCTTGA

Supplementary Table S5: Primary Antibodies

Target	Source	Dilution/Conc.	Application	Manufacturer	Catalog No.
AKT[a]	rabbit	1:1000	WB	Cell Signaling	9272
p-AKT[a] (Ser473)	mouse	1:1000	WB	Cell Signaling	5102
CD31[b]	rat	1:100	IF	BD Pharmingen	550274
GFAP[a]	mouse	1:100	IF	DakoCytomation	M0761
IDH1R132[a]	mouse	1:20	IHC	Dianova	DIA-H09
NDRG1[a]	goat	0.3 µg/ml	WB	Abcam	ab37897
p-NDRG1[a] (Thr346)	rabbit	1:1000	WB	Cell Signaling	5482
Nestin[a]	rabbit	1:200	IF	Merck Millipore	AB5922
NeuN[a,b]	mouse	1:50	IF	Merck Millipore	MAB377
NFκB p65[a]	mouse	1:1000	WB	Cell Signaling	6956
p-NFκB p65[a] (Ser536)	rabbit	1:1000	WB	Cell Signaling	3033
p-p38[a] (Thr180/ Tyr182)	rabbit	1:1000	WB	Cell Signaling	4511
Pathscan Multiplex cocktail I[a]	rabbit	1:200	WB	Cell Signaling	5301
PTEN[a]	rabbit	1:1000<br>1:200	WB<br>IHC	Cell Signaling	9559
α-Tubulin[a]	mouse	1:5000	WB	Sigma Aldrich	T9026
VEGFR-2[a]	rabbit	1:1000<br>1:200	WB, FC<br>IF, IHC	Cell Signaling	2479
VEGFR-2[a]	goat	0.1 µg/mL	FC	R&D Systems	AF357
p-VEGFR-2[a] (Tyr1175)	rabbit	1:1000	WB	Cell Signaling	2478

[a]Antibodies targeting human proteins.
[b]Antibodies targeting murine proteins.
Abbreviations: WB, immunoblot; IF, immunofluorescence; IHC, immunohistochemistry; FC, flow cytometry.

Supplementary Table S6: Secondary Antibodies

Target	Source	Dilution	Application	Manufacturer	Catalog No.
rabbit	donkey	1:3000	WB	GE Healthcare	NA934V
mouse	sheep	1:5000	WB	GE Healthcare	NXA931
rabbit	donkey-Alexa Fluor® 488	1:200	IF, FC	Invitrogen	A-21206
rat	goat-Alexa Fluor® 546	1:500	IF	Invitrogen	A-11081
mouse	donkey-Alexa Fluor® 488	1:500	IF	Jackson Immuno	715–545–155
rabbit	donkey-Alexa Fluor® 647	1:500	IF	Jackson Immuno	711–605–152
goat	donkey-Alexa Fluor® 488	1:200	FC	Invitrogen	A-11055

Abbreviations: WB, immunoblot; IF, immunofluorescence; FC, flow cytometry.
